# Evaluation of EPISEQ SARS-CoV-2 and a Fully Integrated Application to Identify SARS-CoV-2 Variants from Several Next-Generation Sequencing Approaches

**DOI:** 10.3390/v14081674

**Published:** 2022-07-29

**Authors:** Nathalie Mugnier, Aurélien Griffon, Bruno Simon, Maxence Rambaud, Hadrien Regue, Antonin Bal, Gregory Destras, Maud Tournoud, Magali Jaillard, Abel Betraoui, Emmanuelle Santiago, Valérie Cheynet, Alexandre Vignola, Véronique Ligeon, Laurence Josset, Karen Brengel-Pesce

**Affiliations:** 1 BioMérieux SA, 69280 Marcy-l’Étoile, France; nathalie.mugnier@biomerieux.com (N.M.); aurelien.griffon@biomerieux.com (A.G.); maxence.rambaud@biomerieux.com (M.R.); maud.tournoud@biomerieux.com (M.T.); magali.dancette@biomerieux.com (M.J.); abel.betraoui@biomerieux.com (A.B.); emmanuelle.santiago@biomerieux.com (E.S.); valerie.cheynet@biomerieux.com (V.C.); veronique.ligeon@biomerieux.com (V.L.); 2 GenEPII Sequencing Platform, Institut des Agents Infectieux, Hospices Civils de Lyon, 69004 Lyon, France; bruno.simon@chu-lyon.fr (B.S.); hadrien.regue@chu-lyon.fr (H.R.); antonin.bal@chu-lyon.fr (A.B.); gregory.destras@chu-lyon.fr (G.D.); laurence.josset@chu-lyon.fr (L.J.); 3 Joint Research Unit Hospices Civils de Lyon-bioMerieux, Centre Hospitalier Lyon Sud, 69310 Pierre-Benite, France; 4 Biogroup-Oriade-Noviale, 38400 Saint-Martin d’Hères, France; alexandre.vignola@biogroup.fr

**Keywords:** next-generation sequencing, SARS-CoV-2, variant identification, mutation screening, genome assembly, nextstrain clade, pango lineage, bioinformatics

## Abstract

Whole-genome sequencing has become an essential tool for real-time genomic surveillance of severe acute respiratory syndrome coronavirus 2 (SARS-CoV-2) worldwide. The handling of raw next-generation sequencing (NGS) data is a major challenge for sequencing laboratories. We developed an easy-to-use web-based application (EPISEQ SARS-CoV-2) to analyse SARS-CoV-2 NGS data generated on common sequencing platforms using a variety of commercially available reagents. This application performs in one click a quality check, a reference-based genome assembly, and the analysis of the generated consensus sequence as to coverage of the reference genome, mutation screening and variant identification according to the up-to-date Nextstrain clade and Pango lineage. In this study, we validated the EPISEQ SARS-CoV-2 pipeline against a reference pipeline and compared the performance of NGS data generated by different sequencing protocols using EPISEQ SARS-CoV-2. We showed a strong agreement in SARS-CoV-2 clade and lineage identification (>99%) and in spike mutation detection (>99%) between EPISEQ SARS-CoV-2 and the reference pipeline. The comparison of several sequencing approaches using EPISEQ SARS-CoV-2 revealed 100% concordance in clade and lineage classification. It also uncovered reagent-related sequencing issues with a potential impact on SARS-CoV-2 mutation reporting. Altogether, EPISEQ SARS-CoV-2 allows an easy, rapid and reliable analysis of raw NGS data to support the sequencing efforts of laboratories with limited bioinformatics capacity and those willing to accelerate genomic surveillance of SARS-CoV-2.

## 1. Introduction

Whole-genome sequencing of SARS-CoV-2 using next-generation sequencing (NGS) is a powerful tool for studying coronavirus disease 2019 (COVID-19) and tracking the evolution and spread of the virus [1]. Accurate information about the global spread of SARS-CoV-2 is critical to allow an adapted public health response. Multiple protocols have been developed and a huge volume of sequencing data have been generated in the past two years [2,3,4,5]. Sequencing issues associated with data generation and/or interpretation (including contaminations, sequencing errors, assembly errors or other bioinformatics issues) have been reported [1,6,7,8,9,10,11,12], and might lead to erroneous phylogenetic analyses. A main challenge for sequencing laboratories, especially those with limited bioinformatics expertise, lies in the reliable analysis, careful curation and timely deposition of genomic data to public databases such as GISAID [13,14]. A number of bioinformatics tools have been developed, usually requiring some background bioinformatics knowledge [3,15,16,17,18]. Laboratories lacking bioinformatics expertise urgently need the support of an easy-to-use, reliable tool to efficiently analyse and deposit their routine sequencing data into public databases. On the other hand, laboratories wanting to implement routine NGS without saturating their bioinformatics capacity while efficiently contributing to SARS-CoV-2 genomic surveillance need a rapid and integrated bioinformatics analysis tool.

With this in mind, we developed a fully integrated and easy-to-use web-based bioinformatics pipeline (EPISEQ^®^ SARS-CoV-2) to analyse and manage SARS-CoV-2 NGS data generated on common sequencing platforms (including Illumina, San Diego, CA, USA, Oxford Nanopore Technologies, Oxford, UK and ThermoFisher Ion Torrent, Waltham, CA, USA). This pipeline is updated regularly to evolve with the emergence of novel SARS-CoV-2 variants and the implementation of recommended sequencing tools and reagents.

In this study, we compared the EPISEQ SARS-CoV-2 bioinformatics pipeline with a reference pipeline. We also compared the performance of various commercially available SARS-CoV-2 sequencing reagents and platforms on an independent NGS dataset using EPISEQ SARS-CoV-2. We showed that EPISEQ SARS-CoV-2 allows an easy and reliable analysis of raw NGS data to support the sequencing efforts and genome surveillance capacity of laboratories.

## 2. Materials and Methods

### 2.1. Patients and Samples

Nasopharyngeal swab (NPS) samples tested positive for SARS-CoV-2 by quantitative RT-PCR, and cycle threshold (Ct) values ranging from 15 to 30.8 were selected for sequencing in this study. 

Samples used for the validation of the EPISEQ SARS-CoV-2 pipeline vs. a reference method (*n* = 1700) were collected between February 2021 and March 2022 and sequenced as part of random genomic surveillance by the Virology Laboratory of Hospices Civils de Lyon (HCL, France). Investigations were conducted in accordance with the General Data Protection Regulation (Regulation (EU) 2016/679 and Directive 95/46/EC) and the French data protection law (Law 78–17 on 06 January 1978 and Décret 2019–536 on 29 May 2019). 

Samples used for the comparison of kits and sequencing platforms using the EPISEQ SARS-CoV-2 bioinformatics pipeline (*n* = 40) were leftover samples of routine laboratory testing for SARS-CoV-2 infection collected between April 2020 and February 2022, provided by the Virology Laboratory of HCL (Lyon, France), Oriade-Noviale medical laboratories (Saint-Martin d’Hères, France) and Eurofins Biomnis Sample Library (Lyon, France). These samples were sequenced at bioMérieux (Marcy l’Etoile, France), as described below. 

The study was conducted in accordance with the Declaration of Helsinki and followed the standards of Good Clinical Practice. Ethical review and approval were waived for this study, as all samples were collected for regular clinical management, with no additional samples needed for the purpose of the study. Patients were informed of the research and their non-opposition to the use of leftover samples for research purposes was obtained, in accordance with French regulations.

### 2.2. Sequencing

For the EPISEQ SARS-CoV-2 validation study, total nucleic acid was isolated from the NPS samples using the automated MGISP-960 system (MGI Tech Co., Ltd., Shenzhen, China). Eluted nucleic acids were used as inputs for cDNA synthesis. cDNA synthesis and multiplexed amplicon-based whole-viral-genome sequencing was performed using the Illumina COVIDSeq Test (Illumina, San Diego, CA, USA, 20043675), according to the manufacturers’ recommendations, in combination with the ARTIC v3, v4 or v4.1 primer pools (IDT 10006788, 10008554 and 10011442, respectively) developed by the Advancing Real-Time Infection Control (ARTIC) network [19]. Libraries were quantified prior to sequencing using the Qubit dsDNA HS Assay Kit (Invitrogen, Waltham, MA, USA, Q32851) and then 100 bp paired-end sequenced using the NovaSeq 6000 Sequencing System SP flow cell (Illumina, San Diego, CA, USA). Three negative controls were processed per 96-well plate run.

For the kit and sequencing platform comparison study, total nucleic acid was isolated with the NucliSENS *easyMAG* system (bioMérieux, Marcy L’Etoile, France) using the Specific B protocol and an elution volume of 50 μL. Negative control samples (at least one per sequencing run) were generated by processing nuclease-free water as an input sample for nucleic acid extraction. 8 μL eluted nucleic acids (or negative control sample) were used as input for cDNA synthesis. cDNA synthesis and multiplexed amplicon-based whole-viral-genome sequencing were performed using NEBNext^®^ ARTIC kits (New England Biolabs [NEB], Ipswich, MA, USA) and primer pools listed in Table 1, according to the manufacturer’s recommendations [20]. NEBNext^®^ ARTIC kits are designed according to the protocols and primers developed by the ARTIC network [19,21]. Libraries were quantified prior to sequencing using the Qubit dsDNA HS Assay Kit (Invitrogen, Waltham, MA, USA, Q32851). For Illumina sequencing, library quality and size were also evaluated by capillary electrophoresis (Femto Pulse System, Agilent, Santa Clara, CA, USA) using the Ultra Sensitivity NGS Kit (Agilent, Santa Clara, CA, USA, FP-1101-0275). Illumina libraries were denatured and diluted to a final loading concentration of 12 pM following the Illumina MiSeq System Denature and Dilute Libraries Guide (15039740 v10). They were then sequenced on the MiSeq system at 2 × 151 bp using the MiSeq Reagent Kit v3 (600-cycle) (Illumina, San Diego, CA, USA, MS-102-3003) or 2 × 75 bp using the MiSeq Reagent Kit v3 (150-cycle) (Illumina, San Diego, CA, USA MS-102-3001) when NEBNext^®^ ARTIC SARS-CoV-2 FS Library Prep Kit (Illumina, San Diego, CA, USA) (NEB, Ipswich, USA E7658) was used. For Oxford Nanopore Technologies (ONT, Oxford, UK) sequencing, libraries were loaded onto FLO-MIN106D, R9.4.1 Flow Cells and sequenced with a GridION Mk1 instrument (Table 1). 

### 2.3. Sequencing Data Export and Analysis

For the EPISEQ SARS-CoV-2 validation study, reads of Illumina sequencing conducted on the NovaSeq 6000 Sequencing System SP flow cell were first processed for basecalling and demultiplexing using the Illumina DRAGEN Bio-IT Platform. Raw FASTQ reads were then used as input for a reference analysis using the in-house bioinformatics pipeline seqmet (github genEPII) [22], as recently described [23]. Briefly, paired reads were trimmed with cutadapt to remove sequencing adapters and low-quality ends, only keeping reads longer than 30 bp [24]. Alignment to the SARS-CoV-2 reference genome (isolate Wuhan-Hu-1 MN908947.3) was performed using Minimap2 [25]. Mapped reads were processed to remove duplicates tagged by picard, then realigned by abra2 to improve indel detection sensitivity and finally clipped with samtools ampliconclip to remove read ends containing primer sequences [26,27,28]. Variants present at frequencies ≥ 5% were called using freebayes, then decomposed and normalized with vt and filtered with bcftools to eliminate false positives [28,29,30]. Co-infections were detected as previously described [23]. The percentage of coverage of the consensus sequence to the reference genome was calculated and SARS-CoV-2 variant clade and lineage were identified according to the Nextstrain clade and Pango lineage nomenclatures [31,32,33] using Nextclade v1.11.0 and Pangolin v3.1.20, respectively. For a second time, raw FASTQ reads were used as input for analysis by the EPISEQ SARS-CoV-2 application, as described below.

For the kits and sequencing platforms comparison study, basecalling and demultiplexing were conducted using the Real-Time Analysis (RTA) software v1.18.54 (for NGS data generated on the Illumina MiSeq device) or the Guppy software v4.3.4, v5.0.11, v5.0.13 or v5.1.13, as they became available (for NGS data generated on the ONT GridION device). Raw FASTQ reads were then used as input for analysis by the EPISEQ SARS-CoV-2 application. Alignment to the reference genome (isolate Wuhan-Hu-1 MN908947.3), generation of a consensus sequence, percentage of coverage of the reference genome, and identification of amino acid mutations were automatically performed using EPISEQ SARS-CoV-2. SARS-CoV-2 variant clade and lineage were identified in EPISEQ SARS-CoV-2, according to the Nextstrain clade and Pango lineage nomenclatures [31,32,33] using the same version of Nextclade (v1.11.0) and Pangolin (v3.1.20), respectively, as that used for the reference pipeline.

### 2.4. Data Analysis

EPISEQ SARS-CoV-2 was compared to the validated bioinformatics pipeline (github genEPII; [22,23]) set as a reference, using a large set of raw NGS data (*n* = 1700 samples). Genome coverage (% of reference genome) established by EPISEQ SARS-CoV-2 was compared to that determined by the reference method using non-parametric Spearman correlation in GraphPad Prism 5.04. A *p*-value < 0.05 was considered statistically significant. The percentage of coverage was calculated using the following formula: (number of non-ambiguous bases)/29,903 × 100. The percentage of agreement between EPISEQ SARS-CoV-2 and the reference method in clade and lineage assignment and in SARS-CoV-2 amino acid mutation identification was calculated for samples with genome coverage greater than 95% (as determined by the reference method). The Exact Binomial 95% confidence intervals (95% CI) were computed using the SAS Enterprise Guide 8.2 software. In addition, the number of single-nucleotide polymorphisms (SNPs) detected after pairwise alignment of consensus sequences (with >95% genome coverage) generated by EPISEQ SARS-CoV-2 vs. the reference method (between-method SNPs) was evaluated. For concordance analyses, sequence comparisons did not consider regions with undetermined (N) nucleotides and indels (insertions or deletions) in any of the respective consensus sequences.

Following validation against the reference method, EPISEQ SARS-CoV-2 was used to compare the analysis of raw NGS data of SARS-CoV-2-positive samples generated in parallel on two sequencing platforms (Illumina, ONT) using several commercial kits (Table 1). The percentage of reference genome coverage was calculated and depicted as Tukey box plots [34] using GraphPad Prism 5.04. The percentage of concordance between kits and sequencing platforms in clade and lineage assignment was calculated. Variations in nucleotide and amino acid detection between kits and sequencing platforms were recorded and evaluated using heatmaps (designed in R version 3.6.1) and nucleotide sequence alignments (generated using Geneious 10.0.7).

## 3. Results

### 3.1. EPISEQ^®^ SARS-CoV-2 Application

EPISEQ SARS-CoV-2 was developed as a cloud-based application to facilitate the identification and reporting of SARS-CoV-2 variants from raw NGS data (https://www.biomerieux-episeq.com/sars-cov-2, accessed on 10 January 2022). EPISEQ SARS-CoV-2 utilizes FASTQ files generated by various sequencing platforms (Illumina, Oxford Nanopore Technologies, ThermoFisher Ion Torrent) using amplicon-based or target enrichment sequencing protocols according to the ARTIC network recommendations. Following sequence upload, the pipeline performs four successive analyses (Figure S1). 

First, a quality check of the input FASTQ files is performed. It consists of checking the format and integrity of the uploaded files and verifying if enough SARS-CoV-2-related reads are available for analysis using the Fastv public tool [35].

Second, genome assembly is carried out. For Illumina sequencing data, the reads are aligned against the SARS-CoV-2 reference genome (isolate Wuhan-Hu-1 MN908947.3) using bwa (v0.7.17) [36], and automatic detection of the primer kit is performed using a proprietary tool. Primer sequences are then trimmed and a consensus sequence is generated using the ivar (v1.3.1) public tool [37]. For ONT sequencing data, an automatic detection of the primer kit is performed using a proprietary tool before filtering the input reads based on their size to remove potential chimeric reads, aligning the reads on the SARS-CoV-2 reference genome (isolate Wuhan-Hu-1 MN908947.3) using a minimap2 (v2.17) public tool [25], trimming the primers, and creating a consensus sequence according to the ARTIC network bioinformatics protocol [38].

Third, quality controls of the consensus sequence, including its length, the percentage of reference genome coverage, the sequencing depth, the number of ACGT (non-ambiguous) bases, and statistics related to the assembly quality of the spike-coding S gene are performed. 

Fourth, variant identification and mutation screening based on the consensus sequence are conducted. Variants are identified according to the up-to-date Nextstrain clade and Pango lineage nomenclatures using the Nextclade and Pangolin public tools, respectively [31,32,33]. Variants of concerns (VOC) are labelled according to the definitions of the World Health Organization and Centers for Disease Control and Prevention [39]. Mutations are screened in all SARS-CoV-2 genes, including the S gene, using Nextclade [31,33]. 

The complete analysis is performed in one click and takes a few minutes upon NGS FASTQ data upload. As an example, it took 12 min to analyse the 19 omicron samples of the study sequenced on the Illumina platform. Multiple samples can be processed in parallel. Following analysis, a simple report is available for download in portable document format (PDF) (Figure S1). The consensus sequence generated during analysis can be downloaded, and the results can also be exported in batch to a Microsoft Excel file.

### 3.2. Validation of EPISEQ SARS-CoV-2

#### 3.2.1. SARS-CoV-2 Genome Coverage 

**Figure 1:**
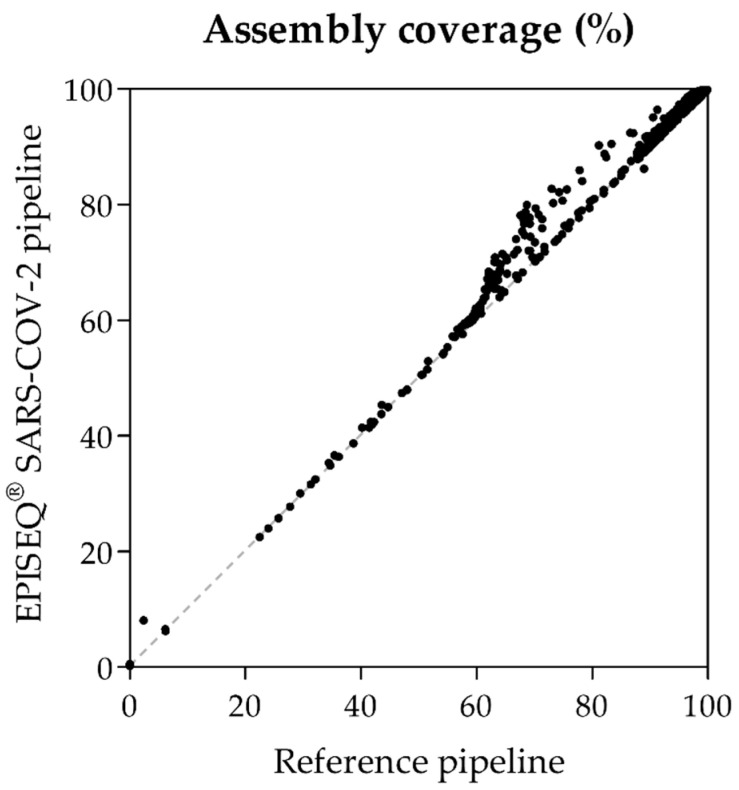
Correlation of the percentage of genome coverage of SARS-CoV-2 sequences evaluated by EPISEQ SARS-CoV-2 vs. the reference method (*n* = 1632). Spearman (non-parametric) correlation coefficient r = 0.883 (95% confidence interval: 0.871–0.893; *p* < 0.0001).

Agreement in sequence analysis by EPISEQ SARS-CoV-2 and the reference method was evaluated using 1700 whole-genome SARS-CoV-2 sequences generated on Illumina NovaSeq 6000. The dataset included sequences of 990 pre-omicron samples sequenced with ARTIC v3 (*n* = 619) and ARTIC v4 (*n* = 371) primer sets and 710 samples of the omicron era sequenced with ARTIC v4.1 primer set. Genome assembly length (expressed in % of the reference genome) of the 1700 samples, as determined by EPISEQ SARS-CoV-2 and the reference method was compared. Following the quality control step by EPISEQ SARS-CoV-2, which considers the percentage of genome coverage, the sequencing depth, and the number of non-ambiguous ACGT bases of the consensus sequence, 68 samples were attributed the status “QC Fail” by EPISEQ SARS-CoV-2. A “QC Fail” status implies that no consensus sequence is generated; these samples were excluded from the comparison. Genome coverage of a total of 1632 sequences calculated by both bioinformatics tools was highly correlated (Figure 1) (Spearman correlation r = 0.883, *p* < 0.0001). 

#### 3.2.2. SARS-CoV-2 Variant Call 

Out of these 1632 sequences, 1362 with a genome coverage > 95% (based on the reference method) were considered to assess the concordance in variant call (Nextstrain clade and Pango lineage) by the EPISEQ SARS-CoV-2 pipeline vs. the reference method (Table 2).

Agreement between both analysis methods to identify SARS-CoV-2 variant clade and lineage over the whole dataset (*n* = 1362) was >99%, ranging from 98.7% to 100.0% depending on the variants investigated and the respective primer pools used (Table 2). The evaluation of the 12 apparent discordant sequences (two for clade and 10 for lineage identification; Table 2) revealed that two sequences were not assigned a clade with the reference method due to a large deletion in the S gene (preventing a comparison with EPISEQ SARS-CoV-2) and that 10 sequences were assigned distinct sub-lineages within the same main lineage by the two pipelines (Table S1). Out of those 10 sequences, slight differences in the percentage of coverage (<1.6%) between both pipelines were observed and four sequences showed one or two single-nucleotide polymorphisms. Samples with differing lineage attributions were concordant in their clade definition and vice-versa (Table S1). Therefore, no major discrepancies were identified between both analysis tools as to clade and lineage assignment. 

#### 3.2.3. SARS-CoV-2 Whole-Genome Consensus Sequence

Genome assemblies performed by the EPISEQ SARS-CoV-2 and the reference pipelines were further compared by evaluating the number of single-nucleotide polymorphisms (SNPs) detected between consensus sequences generated by both pipelines (Table 3). For this nucleotide sequence comparison, regions of the consensus sequences with undetermined (N) nucleotides or indels in either analysis pipeline were excluded.

Altogether, 222/1362 (16.3%) consensus sequences presented 1 to 5 SNPs between both assembly approaches, including 3/527 (0.6%) with 1 SNP for libraries prepared using the ARTIC v3 primer set, 63/316 (19.9%) with 1 or 2 SNPs for the ARTIC v4 primer set, and 156/519 (30.1%) with 1 to 5 SNPs for the ARTIC v4.1 primer set (Table 3). Among the four sequences generated with ARTIC v4.1 showing >2 SNPs, three were identified as resulting from SARS-CoV-2 co-infections [23], likely explaining the higher number of variable nucleotides identified by the two pipelines (3, 4 and 5 SNPs, respectively). Among the 55 (ARTIC v4) and 137 (ARTIC v4.1) sequences with 1 SNP (Table 3), a small proportion (8/55 [14.5%] for ARTIC v4 and 13/137 [9.5%] for ARTIC v4.1) was linked to poor quality sequences, notably low sequencing depth (<13 reads), suggesting sequence inaccuracy rather than true polymorphism between both consensus sequences. The majority of sequences with 1 SNP (47/55 [85.5%] for ARTIC v4 and 124/137 [90.5%] for ARTIC v4.1) corresponded to an apparent polymorphism (two nucleotides identified in approximate equal proportions amidst the generated reads and randomly assigned to the consensus sequence based on a majority rule specific to each bioinformatic tool) at three main nucleotide positions in the SARS-CoV-2 genome: C8829A, T8835C and T15521A (position relative to the reference genome). More precisely, out of the 47 ARTIC v4 sequences with 1 SNP, 19 showed a polymorphism at position 8829, 19 at position 8835 and 9 at position 15521. Similarly, out of the 124 ARTIC v4.1 sequences with 1 SNP, 2 showed a polymorphism at position 8829, 77 at position 8835 and 45 at position 15521. These apparent polymorphisms were also identified among the ARTIC v4 and v4.1 sequences with 2 SNPs (Table 3). Nucleotide polymorphisms C8829A, T8835C and T15521A lie within amplicons (not primer-annealing regions), and predict the following amino acid mutations: ORF1a:A2855D, ORF1a:V2857A and ORF1b:F685Y, respectively. Polymorphisms T8835C and T15521A have been reported as sequencing artefacts associated with ARTIC v4 and v4.1 primer schemes resulting from mispriming events within amplicons 29 and 51, respectively [40]. To our knowledge, the less frequent C8829A apparent polymorphism has not been reported to date as a sequencing artefact. 

#### 3.2.4. SARS-CoV-2 Spike Protein Mutations

Concordance in the detection of amino acid mutations within the protein spike by both pipelines was also examined. Regions with undetermined sequences in either analysis pipeline were excluded from the comparison. Amino acid identified by both pipelines showed a strong agreement (>99% over all sequencing data), ranging from 98.3% for sequences generated by ARTIC v4.1 to 100% for sequences generated by ARTIC v3 (Table 4). Each of the 10 discordances observed with ARTIC v4 or v4.1 corresponded to polymorphisms in roughly equal proportions among reads, which were designated as consensus in one of the pipelines.

Altogether, sequence analyses provided by EPISEQ SARS-CoV-2 as to genome assembly, clade and lineage classification, and SNP identification were in strong agreement with those provided by the reference method. Evaluation of discordances also demonstrated that EPISEQ SARS-CoV-2 performed at least as well as the reference method.

### 3.3. Comparative Performance of Sequencing Platforms and Kits Using EPISEQ SARS-CoV-2

We next evaluated the compatibility of the EPISEQ SARS-CoV-2 tool for the analysis of data generated by commonly used sequencing platforms and reagents. We used EPISEQ SARS-CoV-2 to compare the sequencing results obtained on two sequencing platforms (Illumina MiSeq and ONT GridION Mk1) using different commercial kits and primer pools (Table 1). Altogether, 40 SARS-CoV-2-positive samples covering a broad range of Ct values (15.0–30.8) and including pre-omicron (*n* = 21) and omicron (*n* = 19) SARS-CoV-2 variants were selected for this analysis, thus generating a total of 244 raw sequencing results (Tables S2 and S3).

#### 3.3.1. SARS-CoV-2 Genome Coverage 

**Figure 2:**
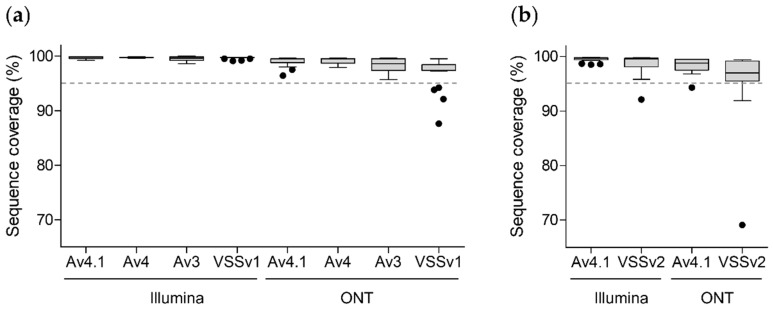
Percentage of reference genome coverage determined by EPISEQ SARS-CoV-2 upon whole-genome sequencing with different kits and sequencing platforms (Table 1). (**a**) Pre-omicron SARS-CoV-2-positive samples (including seven 20A-G (EU1), four alpha, two beta, one gamma, six delta and one Eta SARS-CoV-2 variants; *n* = 21) were sequenced using four different commercial kits and primer pools (ARTIC v3, v4, v4.1 and VSS v1) on two NGS platforms (Illumina MiSeq and ONT GridION), generating 168 sequencing results. (**b**) Omicron-positive SARS-CoV-2 samples (including nine BA.1 and 10 BA.2 omicron sub-variants; *n* = 19) were sequenced using two different commercial kits and primer pools (ARTIC v4.1 and VSS v2) on the same two NGS platforms (Illumina MiSeq and ONT GridION), generating 76 sequencing results. A total of 244 raw NGS data were generated and analysed using EPISEQ SARS-CoV-2. The dashed line indicates 95% coverage (quality control criteria). Abbreviations: Av4.1, ARTIC kit version 4.1; Av4, ARTIC kit version 4; Av3, ARTIC kit version 3; Illumina, San Diego, USA Illumina sequencing; ONT, Oxford, UK Oxford Nanopore Technologies sequencing; VSSv1, VarSkip Short kit version 1; VSSv2, VarSkip Short kit version 2.

The quality of the 244 NGS data was evaluated by calculating the proportion of genome coverage with EPISEQ SARS-CoV-2 (Figure 2). 235/244 (96.3%) NGS results showed a coverage of the reference genome >95%, with a median coverage ranging from 99.6% to 99.8% on the Illumina platform and from 97.0% to 99.5% on the ONT platform (Figure 2a,b). Out of the nine NGS results with a coverage <95%, seven originated from sequencing on ONT using VSS (v1 or v2) primer sets, one from sequencing on Illumina using VSS v2 primer set, and one from sequencing on ONT using ARTIC v4.1 primer set (Figure 2). 

#### 3.3.2. SARS-CoV-2 Variant Call 

The analysis of the concordance in clade and lineage identification by EPISEQ SARS-CoV-2 between the different sequencing approaches revealed a 100% concordance over the 40 analysed samples (Table 5) and 243/244 NGS data (Tables S2 and S3). One sequencing result with very low genome coverage (69.1%) could not be assigned a Pango lineage by EPISEQ SARS-CoV-2, although the correct clade was attributed (sample 22; Table S3, yellow field).

#### 3.3.3. SARS-CoV-2 Amino Acid and Nucleotide Mutations

As to amino acid mutation identification by EPISEQ SARS-CoV-2 within and outside the spike protein, a partial concordance (18/40 [45.0%] within spike, 28/40 [70.0%] outside spike) between the sequencing approaches was observed, as expected from the comparison of kits with different amplification specificities (ARTIC v3, v4, v4.1 and VSS v1 and v2) (Tables S2 and S3).

**Figure 3:**
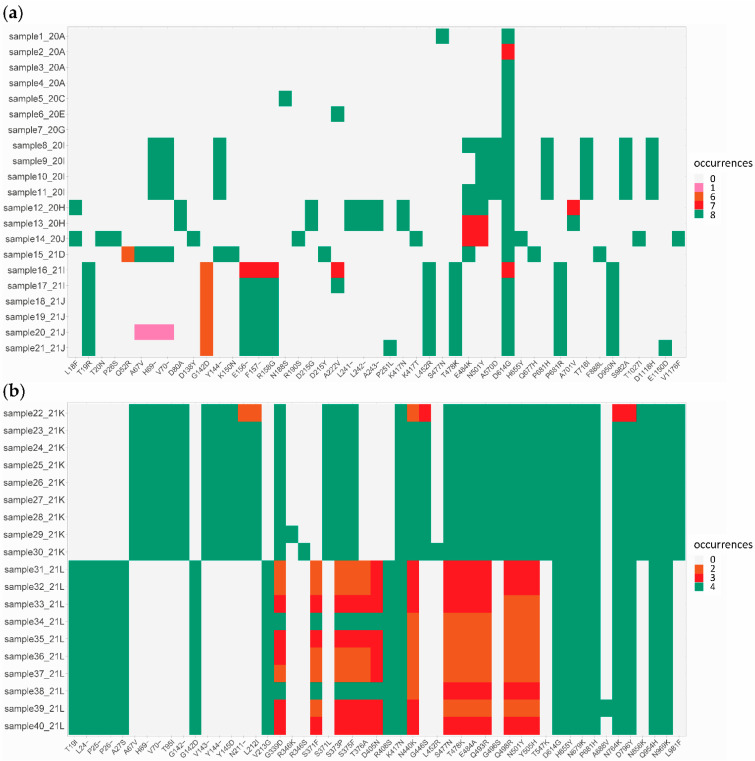
Spike mutations identified by EPISEQ SARS-CoV-2. (**a**) Pre-omicron SARS-CoV-2 variants (including 4 alpha, 2 beta, 1 gamma, 6 delta and 8 other SARS-CoV-2 variants; *n* = 21); (**b**) SARS-CoV-2 omicron variants (including 9 BA.1 [21K] and 10 BA.2 [21L] sub-variants; *n* = 19). Concordance in mutation detection between kits and sequencing platforms is shown in dark green (mutation detected in all eight (**a**) or four (**b**) conditions) and light grey (no mutation detected in all eight (**a**) or four (**b**) conditions). Other colours (pink, orange and red) represent mutations detected with some but not all kit/sequencer combinations, thus indicating a discordance in identified mutations (see Tables S2 and S3 for details).

A detailed analysis of concordant and discordant mutations within spike showed an overall good concordance (Figure 3, dark green and light grey) between all approaches for pre-omicron samples using ARTIC v3, v4, v4.1 and VSS v1 primers (Figure 3a, samples 1 to 21) and for omicron BA.1 samples using ARTIC v4.1 and VSS v2 primers (Figure 3b, samples 22 to 30), except for one sample with low genome coverage (Figure 3b, sample 22). Discordant results (Figure 3, pink, orange and red) were mainly due to the use of outdated primer pools, notably ARTIC v3 vs. v4.1 for the sequencing of delta variants (Figure 3a, samples 16 to 21) or to differences in sequencing performance between ARTIC v4.1 and VSS v2 for the sequencing of omicron BA.2 variants, especially between amino acids 339 and 505 (Figure 3b, samples 31 to 40). In these BA.2 variants, the differences also appeared to be sample dependent.

**Figure 4:**
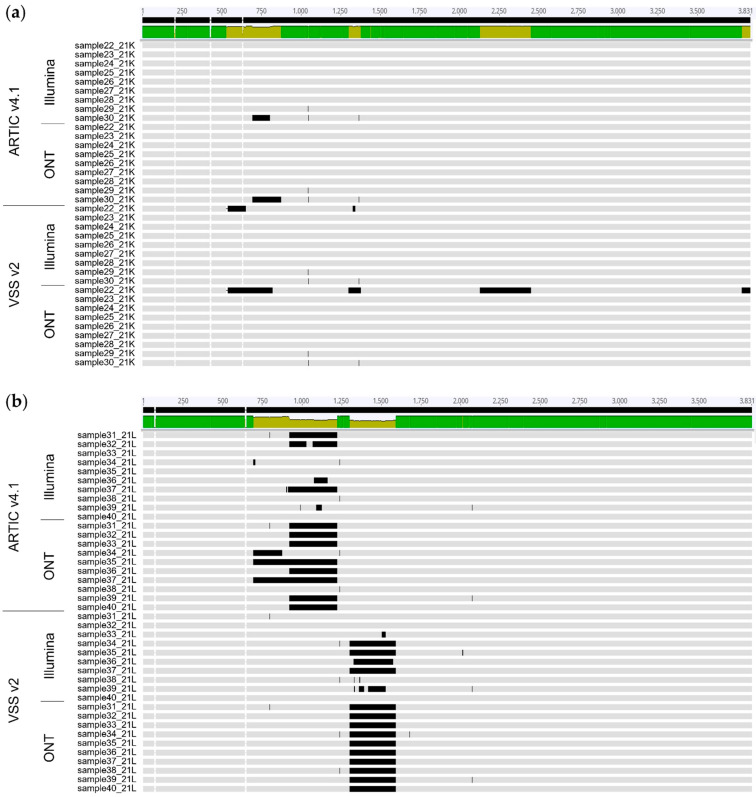
Genomic assembly of sequences of the S gene of SARS-CoV-2 omicron variants by EPISEQ SARS-CoV-2 following sequencing with two different kits (based on ARTIC v4.1 and VSS v2) and on two different platforms (Illumina and ONT). (**a**) SARS-CoV-2 Omicron BA.1 (21K) variants (*n* = 9). (**b**) SARS-CoV-2 omicron BA.2 (21L) variants (*n* = 10). Horizontal black bars represent undetermined bases (N). Legend of sequence alignment (performed with Geneious 10.0.7): bright green, all aligned sequences are identical; light green, some aligned sequences differ (mismatch, undetermined nucleotides or deletions in some sequences). The genomic region covering nucleotides ~700 to 1700 shows sequencing gaps using ARTIC v4.1 (between nucleotides ~700 to 1250, overlapping amplicon 75) and VSS v2 (between nucleotides ~1300 to 1700, overlapping amplicon 57).

Differences in performance between ARTIC v4.1 and VSS v2 primers for the sequencing of omicron BA.2 variants were confirmed by analysing the alignment of the respective S gene nucleotide sequences (Figure 4). Sequences generated using ARTIC v4.1 often showed gaps of undetermined sequences between nucleotides ~700 and 1250 (overlapping amplicon 75), while sequences produced using VSS v2 showed gaps between nucleotides ~1300 and 1700 (overlapping amplicon 57) (Figure 4b, horizontal black bars). These amplicon dropouts over amplicons 75 (ARTIC v4.1) and 57 (VSS v2) were likely due to sequencing failures due to mutations within the BA.2 variant that overlap primer 75R (ARTIC v4.1; two mutations at positions 2 and 7 of primer 75R) and 57L (VSS v2; one mutation at position 27 of primer 57L), respectively, as recently reported [41]. Thus, both ARTIC v4.1 and VSS v2 primer pools presented flaws in accurately sequencing the S gene of the omicron BA.2 variants. These flaws explain the discordant results in amino acid mutations detected by EPISEQ SARS-CoV-2 between the sequencing approaches (Figure 3b, orange and red colours, amino acids 339 to 505). Sequencing gaps were rarely observed using the same primers on samples of omicron BA.1 variants (Figure 4a), except for sample 22 sequenced with VSS v2 on the ONT device, in line with the low genomic coverage described earlier (Table S3 and Figure 3b, orange and red colours).

Finally, considering the mispriming artefacts observed in the validation phase (T8835C, T15521A and possibly C8829A), which was based on NGS data obtained using a different protocol (Illumina COVIDSeq Test on NovaSeq 6000 sequencer), we evaluated the 244 sequencing results obtained in this kit comparison analysis for the presence of polymorphisms at nucleotide positions 8829, 8835 and 15521. None of the 244 generated consensus sequences showed the C8829A, T8835C or T15521A apparent polymorphisms. Coincidently, eight BA.2 omicron samples out of the 40 analysed samples had also been sequenced using the reference protocol (Illumina COVIDSeq Test on NovaSeq 6000, using ARTIC v4.1 primers). Interestingly, of these eight sequences, one presented the T15521A artefact mutation. 

## 4. Discussion

This study describes the validation of EPISEQ SARS-CoV-2, an easy-to-use and integrative (“one-click”) web-based application developed for sequencing laboratories lacking bioinformatics capacity or wishing to speed up SARS-CoV-2 genomic surveillance without saturating their internal bioinformatics capacity. EPISEQ SARS-CoV-2 can analyse raw NGS data generated by different sequencing platforms (Illumina, ONT) within minutes using a variety of kits and primer pools.

We showed that EPISEQ SARS-CoV-2 provides results comparable to those of a reference in-house bioinformatics pipeline in terms of genome coverage (Spearman correlation coefficient r = 0.883; *p* < 0.0001), Nextstrain clade and Pango lineage classifications (>99% concordance), and amino acid substitution identification (>99% concordance within the spike protein), over 1362 NGS data covering alpha to omicron SARS-CoV-2 variants. Interestingly, the comparison of the nucleotide consensus sequences generated by both pipelines upon sequencing with ARTIC v4 and v4.1 revealed the presence of apparent SNPs (T8835C, T15521A) actually resulting from sequencing errors (mispriming artefacts) frequently detected with ARTIC v4 and v4.1 [40]. These sequencing artefacts were observed in 11–22% (T8835C) and 43–47% (T15521A) of the validation dataset (depending on the pipeline used), thus representing an important proportion of artefactual mutations. Several types of sequencing artefacts resulting from mispriming, cross-primer dimerisation or reduced coverage due to amplicon dropout have been described [11,40,41,42]. These sequencing errors can lead to fallacious mutation reporting and distort phylogenetic trees. They can also lead to erroneous biological interpretations, as illustrated by the misinterpretation of mutation G142D being associated with a higher SARS-CoV-2 viral load [11]. Interestingly, in our study, detection of the mispriming artefacts T8835C and T15521A not only depended on the use of ARTIC v4 and v4.1 primers but also seemed to depend on the sequencing protocols (Illumina COVIDSeq Test vs. NEBNext^®^ ARTIC SARS-CoV-2), regardless of the bioinformatics pipeline used. Similarly to our kit comparison analysis, Lambisia et al. reported that they did not detect T8835C and T15521A SNPs using ARTIC v4 primers with their sequencing protocol [43]. Thus, differences in wet lab protocols should be carefully examined regarding the possible occurrence of systematic sequencing errors. In addition, sequence analysis solutions such as error pre-screening, amplicon size filtering and problematic site masking should be considered to avoid erroneous mutation reporting [40,44].

The comparison of 40 NGS data generated by a variety of sequencing approaches (ARTIC and VSS kits on Illumina and ONT platforms) using EPISEQ SARS-CoV-2 revealed a perfect concordance in Nextstrain clade and Pango lineage classifications. It also allowed for the identification of differences in performance in terms of genome coverage and in the identification of mutations (within and outside spike). Such differences were in part expected due to established differences in specificities associated with some of the primer pools (notably between ARTIC v3, v4 and v4.1) [45,46]. In addition, this analysis identified flaws in ARTIC v4.1 and VSS v2 primers for the sequencing of two distinct regions of the S gene of the omicron BA.2 subvariant due to amplicon dropouts that had been previously reported [41].

EPISEQ SARS-CoV-2 is a “one-click” application that allows a rapid and reliable analysis of SARS-CoV-2 NGS data. The results of the analysis, which are essential for proper genomic surveillance of SARS-CoV-2 (variant calls, mutation identification), can then be exported in a simple report (Figure S1). This approach is particularly important for small sequencing laboratories with limited bioinformatics capacity and those needing to improve genomic surveillance and is thus highly relevant in times of pandemics. In comparison, the bioinformatics pipelines provided with the respective sequencing platforms (Dynamic Read Analysis for GENomics [DRAGEN] Bio-IT Platform and DRAGEN COVID Lineage application, Illumina; EPI2ME cloud-based analysis platform Fastq QC  +  ARTIC  +  NextClade, ONT), albeit reliable, are more complex to use and their results are more difficult to locate, extract and interpret for a non-specialist in bioinformatics. For instance, the configuration of these pipelines requires the user to specify analysis parameters (e.g., primers used), the analysis provides loads of details, sometimes in separate and large tables, and some results cannot be exported in a simple file, all of which might confuse a non-specialist and possibly be more error-prone in routine analyses. On the other hand, these platforms allow data visualisation (e.g., phylogenetic tree), which is not provided by EPISEQ SARS-CoV-2. Additionally, EPISEQ SARS-CoV-2 cannot report complex situations, such as co-infections, as opposed to the reference pipeline used in this study [23].

A strength of this study is the use of a large number of samples (1362) for the validation of EPISEQ SARS-CoV-2 against a reference bioinformatics method, the choice of samples covering a broad range of past and present SARS-CoV-2 variants, and the comparison of NGS data generated in parallel on two sequencing platforms (Illumina MiSeq and ONT GridION Mk1) using a total of 5 different kits (ARTIC v3, v4, v4.1 and VSS v1, v2), thus comparing up to 4 or 8 experimental combinations, depending on the variants investigated (omicron or pre-omicron, respectively). A possible limitation of this study is that the performance of EPISEQ SARS-CoV-2 to identify indels was not evaluated. In addition, this study focused on amplicon-based sequencing methods, which are most commonly used in the current era of ongoing SARS-CoV-2 genomic surveillance, and on two sequencing platforms (Illumina and ONT). However, preliminary data indicated that EPISEQ SARS-CoV-2 is compatible with target-enrichment sequencing approaches and that it can analyse NGS data generated by additional sequencing platforms, such as ThermoFisher Ion Torrent (not shown). Thus, in addition to being regularly updated as new SARS-CoV-2 variants emerge, EPISEQ SARS-CoV-2 has been conceived to evolve with the implementation of novel sequencing approaches and reagents according to official recommendations. 

## 5. Conclusions

EPISEQ SARS-CoV-2 is a reliable and easy-to-use web-based application conceived to support the analysis of SARS-CoV-2 NGS data and the reporting of identified mutations by laboratories with limited bioinformatics skills. The platform is updated weekly to evolve with the reporting of new SARS-CoV-2 Nextstrain clades, Pango lineages, and VOC. The application is also conceived to evolve with the implementation of new sequencing tools. 

## Figures and Tables

**Table 1:** Sequencers and reagents used for the kits and sequencing platform comparison study.

Sequencer	Primer Pool	Kits
MiSeq (Illumina)	ARTIC v3	NEBNext^®^ ARTIC SARS-CoV-2 Library Prep Kit (Illumina) (NEB, E7650)
	ARTIC v4	NEBNext^®^ ARTIC SARS-CoV-2 Library Prep Kit (Illumina) (NEB, E7650); ARTIC V4 NCOV-2019 Panel (IDT, 10008554)
	ARTIC v4.1	NEBNext^®^ ARTIC SARS-CoV-2 FS Library Prep Kit (Illumina) (NEB, E7658); ARTIC V4.1 NCOV-2019 Panel (IDT, 10011442)
	VSS v1	NEBNext^®^ ARTIC SARS-CoV-2 FS Library Prep Kit (Illumina) (NEB, E7658)
	VSS v2	NEBNext^®^ ARTIC SARS-CoV-2 FS Library Prep Kit (Illumina) (NEB, E7658)
GridION Mk1 (Oxford Nanopore Technologies)	ARTIC v3	NEBNext^®^ ARTIC SARS-CoV-2 Companion Kit (ONT) (NEB, E7660)
	ARTIC v4	NEBNext^®^ ARTIC SARS-CoV-2 Companion Kit (ONT) (NEB, E7660); ARTIC V4 NCOV-2019 Panel (IDT, 10008554)
	ARTIC v4.1	NEBNext^®^ ARTIC SARS-CoV-2 Companion Kit (ONT) (NEB, E7660); ARTIC V4.1 NCOV-2019 Panel (IDT, 10011442)
	VSS v1	NEBNext^®^ ARTIC SARS-CoV-2 Companion Kit (ONT) (NEB, E7660)
	VSS v2	NEBNext^®^ ARTIC SARS-CoV-2 Companion Kit (ONT) (NEB, E7660)

Abbreviations: IDT, Integrated DNA Technologies; NEB, New England Biolabs; ONT, Oxford Nanopore Technologies; VSS, VarSkip Short.

**Table 2:** Agreement of SARS-CoV-2 sequence analyses by the EPISEQ SARS-CoV-2 vs. the reference pipeline as to clade and lineage identification (samples with >95% coverage based on the reference method; *n* = 1362).

	Nextstrain Clade		Pango Lineage	
Sequencing Kit	*n/N* ^1^	% [95% CI]	*n/N* ^1^	% [95% CI]
ARTIC v3	527/527 ^2^	100.0% [99.3–100.0]	525/527	99.6% [98.6–99.9]
ARTIC v4	316/316 ^3^	100.0% [98.8–100.0]	315/316	99.7% [98.3–99.9]
ARTIC v4.1	517/519 ^4^	99.6% [98.6–99.9]	512/519	98.7% [97.2–99.5]
Total	1360/1362	99.9% [99.5–100.0]	1352/1362	99.3% [98.7–99.7]

^1^ *n/N* is the ratio of the number of sequences attributed the same clade or lineage, respectively, by both bioinformatics pipelines to the number of sequences analysed. ^2^ Clade distribution (*n* = 527): 19B, 4; 20A, 80; 20B, 10; 20C, 1; 20D, 1; 20E (EU1), 26; 20H (Beta, V2), 16; 20I (Alpha, V1), 388; 21D (Eta), 1. ^3^ Clade distribution (*n* = 316): 20A, 5; 20B, 1; 20H (Beta, V2), 2; 20I (Alpha, V1), 54; 21I (Delta), 16; 21J (Delta), 238. ^4^ Clade distribution (*n* = 519): 20A, 7; 20H (Beta, V2), 5; 21I (Delta), 3; 21J (Delta), 43; 21K (Omicron), 204; 21L (Omicron), 257. Abbreviation: CI, confidence interval.

**Table 3:** Proportion of SARS-CoV-2 sequences presenting single-nucleotide polymorphisms (SNPs) between consensus sequence assemblies generated by EPISEQ SARS-CoV-2 and the reference method.

Sequencing Kit	0 SNP *n/N* ^1^ (%)	1 SNP *n/N* ^1^ (%)	2 SNPs *n/N* ^1^ (%)	>2 SNPs *n/N* ^1^ (%)
ARTIC v3	524/527 (99.4%)	3/527 (0.6%)	0/527 (0.0%)	0/527 (0.0%)
ARTIC v4	253/316 (80.1%)	55/316 (17.4%)	8/316 (2.5%)	0/316 (0.0%)
ARTIC v4.1	363/519 (69.9%)	137/519 (26.4%)	15/519 (2.9%)	4/519 (0.8%)
Total	1140/1362 (83.7%)	195/1362 (14.3%)	23/1362 (1.7%)	4/1362 (0.3%)

^1^ *n/N* is the ratio of the number of consensus sequences with the indicated number of SNPs (0, 1, 2 or >2, respectively) between analyses by both bioinformatics pipelines to the number of consensus sequences analysed.

**Table 4:** Agreement of SARS-CoV-2 sequence analyses by the EPISEQ SARS-CoV-2 vs. the reference pipeline as to amino acid mutation identification (samples with >95% coverage by reference method; *n* = 1362).

Sequencing Kit	Spike Mutations, *n/N* ^1^ (%)
ARTIC v3	527/527 (100.0%)
ARTIC v4	315/316 (99.7%)
ARTIC v4.1	510/519 (98.3%)
Total	1352/1362 (99.3%)

^1^*n/N* is the ratio of the number of sequences with the same amino acid mutations identified by both bioinformatics pipelines within the spike protein to the number of sequences analysed.

**Table 5:** Concordance of sequencing results of SARS-CoV-2-positive samples generated by different kits and sequencing platforms and analysed using the EPISEQ SARS-CoV-2 pipeline.

SARS-CoV-2 Samples	Nextstrain Clade	Pango Lineage
Pre-omicron variants ^1^	21/21 (100.0%)	21/21 (100.0%)
Omicron variants ^2^	19/19 (100.0%)	19/19 (100.0%)
Total	40/40 (100.0%)	40/40 (100.0%)

^1^ Samples (*n* = 21) sequenced using primers ARTIC v4.1, ARTIC v4, ARTIC v3 and VSS v1 on the Illumina and ONT platforms (*n* = 168 output results); ^2^ samples (*n* = 19) sequenced using primers ARTIC v4.1 and VSS v2 on the Illumina and ONT platforms (*n* = 76 output results).

## Data Availability

The data presented in this study are available on request from the corresponding author. The data are not publicly available due to their containing information that could compromise the privacy of research participants.

## Supplementary Materials

The following supporting information can be downloaded at: https://www.mdpi.com/article/10.3390/v14081674/s1, Figure S1: description of EPISEQ SARS-CoV-2 pipeline workflow (**a**), result display (**b**) and sample report (**c**); Table S1: Description of the 12 (out of 1362) samples discordant in their Nextstrain clade or Pango lineage assignment between EPISEQ SARS-CoV-2 and the reference pipeline; Table S2: Evaluation of SARS-CoV-2 sequencing results with the EPISEQ SARS-CoV-2 pipeline (pre-omicron variants; *n* = 21 samples); Table S3: Evaluation of SARS-CoV-2 sequencing results with the EPISEQ SARS-CoV-2 pipeline (omicron variants; *n* = 19 samples).

## References

[1] Worobey M., Pekar J., Larsen B.B., Nelson M.I., Hill V., Joy J.B., Rambaut A., Suchard M.A., Wertheim J.O., Lemey P. (2020). The Emergence of SARS-CoV-2 in Europe and North America. Science.

[2] Charre C., Ginevra C., Sabatier M., Regue H., Destras G., Brun S., Burfin G., Scholtes C., Morfin F., Valette M. (2020). Evaluation of NGS-Based Approaches for SARS-CoV-2 Whole Genome Characterisation. Virus Evol..

[3] Chiara M., D’Erchia A.M., Gissi C., Manzari C., Parisi A., Resta N., Zambelli F., Picardi E., Pavesi G., Horner D.S. (2021). Next Generation Sequencing of SARS-CoV-2 Genomes: Challenges, Applications and Opportunities. Brief. Bioinform..

[4] Liu T., Chen Z., Chen W., Chen X., Hosseini M., Yang Z., Li J., Ho D., Turay D., Gheorghe C.P. (2021). A Benchmarking Study of SARS-CoV-2 Whole-Genome Sequencing Protocols Using COVID-19 Patient Samples. iScience.

[5] Wang M., Fu A., Hu B., Tong Y., Liu R., Liu Z., Gu J., Xiang B., Liu J., Jiang W. (2020). Nanopore Targeted Sequencing for the Accurate and Comprehensive Detection of SARS-CoV-2 and Other Respiratory Viruses. Small.

[6] De Maio N., Walker C., Borges R., Weilguny L., Slodkowicz G., Goldman N. Issues with SARS-CoV-2 Sequencing Data. https://virological.org/t/issues-with-sars-cov-2-sequencing-data/473.

[7] Yutao F. SARS-CoV-2 Samples from Same Early COVID-19 Patients Were Sequenced Repeatedly with Errors Distorting Phylogenetic Trees. https://virological.org/t/sars-cov-2-samples-from-same-early-covid-19-patients-were-sequenced-repeatedly-with-errors-distorting-phylogenetic-trees/434.

[8] Kreier F. (2022). Deltacron: The Story of the Variant That Wasn’t. Nature.

[9] Maxmen A. (2021). Omicron Blindspots: Why It’s Hard to Track Coronavirus Variants. Nature.

[10] Pekar J., Parker E., Havens J.L., Suchard M.A., Andersen K.G., Moshiri N., Worobey M., Rambaut A., Wertheim J.O. Evidence Against the Veracity of SARS-CoV-2 Genomes Intermediate between Lineages A and B. https://virological.org/t/evidence-against-the-veracity-of-sars-cov-2-genomes-intermediate-between-lineages-a-and-b/754.

[11] Sanderson T., Barrett J.C. (2021). Variation at Spike Position 142 in SARS-CoV-2 Delta Genomes Is a Technical Artifact Caused by Dropout of a Sequencing Amplicon. Wellcome Open Res..

[12] Davis J.J., Long S.W., Christensen P.A., Olsen R.J., Olson R., Shukla M., Subedi S., Stevens R., Musser J.M. (2021). Analysis of the ARTIC Version 3 and Version 4 SARS-CoV-2 Primers and Their Impact on the Detection of the G142D Amino Acid Substitution in the Spike Protein. Microbiol. Spectr..

[13] GISAID—HCov19 Variants. https://www.gisaid.org/hcov19-variants//.

[14] Shu Y., McCauley J. (2017). GISAID: Global Initiative on Sharing All Influenza Data—From Vision to Reality. Eurosurveill.

[15] Tilloy V., Cuzin P., Leroi L., Guérin E., Durand P., Alain S. (2022). ASPICov: An Automated Pipeline for Identification of SARS-Cov2 Nucleotidic Variants. PLoS ONE.

[16] Wagner D.D., Marine R.L., Ramos E., Ng T.F.F., Castro C.J., Okomo-Adhiambo M., Harvey K., Doho G., Kelly R., Jain Y. (2022). VPipe: An Automated Bioinformatics Platform for Assembly and Management of Viral Next-Generation Sequencing Data. Microbiol. Spectr..

[17] Rueca M., Giombini E., Messina F., Bartolini B., Di Caro A., Capobianchi M.R., Gruber C.E. (2022). The Easy-to-Use SARS-CoV-2 Assembler for Genome Sequencing: Development Study. JMIR Bioinform. Biotech..

[18] Farkas C., Mella A., Turgeon M., Haigh J.J. (2021). A Novel SARS-CoV-2 Viral Sequence Bioinformatic Pipeline Has Found Genetic Evidence That the Viral 3′ Untranslated Region (UTR) Is Evolving and Generating Increased Viral Diversity. Front. Microbiol..

[19] Advancing Real-Time Infection Control (ARTIC) Network—ARTIC Network. https://artic.network/.

[20] Gautreau I. NEBNext^®^ ARTIC Protocols Collection. https://www.protocols.io/view/nebnext-artic-protocols-collection-bw2apgae.

[21] Quick J. NCoV-2019 Sequencing Protocol v2 (GunIt). https://www.protocols.io/view/ncov-2019-sequencing-protocol-v2-bdp7i5rn.

[22] Genepii Seqmet. https://github.com/genepii/seqmet.

[23] Bal A., Simon B., Destras G., Chalvignac R., Semanas Q., Oblette A., Queromes G., Fanget R., Regue H., Morfin F. (2022). Detection and Prevalence of SARS-CoV-2 Co-Infections during the Omicron Variant Circulation, France, December 2021–February 2022. Medrxiv.

[24] Martin M. (2011). Cutadapt Removes Adapter Sequences from High-Throughput Sequencing Reads. EMBnet. J..

[25] Li H. (2018). Minimap2: Pairwise Alignment for Nucleotide Sequences. Bioinform..

[26] Picard Tools—By Broad Institute. http://broadinstitute.github.io/picard/.

[27] Mose L.E., Perou C.M., Parker J.S. (2019). Improved Indel Detection in DNA and RNA via Realignment with ABRA2. Bioinform..

[28] Danecek P., Bonfield J.K., Liddle J., Marshall J., Ohan V., Pollard M.O., Whitwham A., Keane T., McCarthy S.A., Davies R.M. (2021). Twelve Years of SAMtools and BCFtools. GigaScience.

[29] Garrison E., Marth G. (2012). Haplotype-Based Variant Detection from Short-Read Sequencing. arXiv.

[30] Tan A., Abecasis G.R., Kang H.M. (2015). Unified Representation of Genetic Variants. Bioinform.

[31] Nextstrain—Nextclade. https://github.com/nextstrain/nextclade.

[32] Centre for Genomic Pathogen Surveillance—Phylogenetic Assignment of Named Global Outbreak LINeages (Pangolin). https://github.com/cov-lineages/pangolin.

[33] Aksamentov I., Roemer C., Hodcroft E.B., Neher R.A. (2021). Nextclade: Clade Assignment, Mutation Calling and Quality Control for Viral Genomes. J. Open Source Softw..

[34] Krzywinski M., Altman N. (2014). Visualizing Samples with Box Plots. Nat. Methods.

[35] Chen S., He C., Li Y., Li Z., Melançon C.E. (2021). A Computational Toolset for Rapid Identification of SARS-CoV-2, Other Viruses and Microorganisms from Sequencing Data. Brief. Bioinform..

[36] Li H. (2013). Aligning Sequence Reads, Clone Sequences and Assembly Contigs with BWA-MEM. arXiv.

[37] Grubaugh N.D., Gangavarapu K., Quick J., Matteson N.L., De Jesus J.G., Main B.J., Tan A.L., Paul L.M., Brackney D.E., Grewal S. (2019). An Amplicon-Based Sequencing Framework for Accurately Measuring Intrahost Virus Diversity Using PrimalSeq and IVar. Genome. Biol..

[38] Loman N., Rowe W., Rambaut A. ARTIC-NCoV-BioinformaticsSOP-v1.1.0. https://artic.network/ncov-2019/ncov2019-bioinformatics-sop.html.

[39] ECDC SARS-CoV-2 Variants of Concern (VOC). https://www.ecdc.europa.eu/en/covid-19/variants-concern.

[40] Sanderson T., De Maio N., Hinrichs A.S., de Bernardi Schneider A., Walker C., Goldman N., Turakhia Y., Lanfear R., Corbett-Detig R. Issues with SARS-CoV-2 Sequencing Data—Systematic Errors Associated with Some Implementations of ARTIC V4 and a Fast Workflow to Prescreen Samples for New Problematic Sites. https://virological.org/t/issues-with-sars-cov-2-sequencing-data/473.

[41] New England Biolabs SARS-CoV-2 Lineage Variant Summary. https://primer-monitor.neb.com/lineages.

[42] Wilkinson S., Groves N., Quick J. Loman Erroneous Mutations Associated with 64_L-60_R Primer-Dimer in ARTIC 4/4.1—Laboratory. https://community.artic.network/t/erroneous-mutations-associated-with-64-l-60-r-primer-dimer-in-artic-4-4-1/419.

[43] Lambisia A.W., Mohammed K.S., Makori T.O., Ndwiga L., Mburu M.W., Morobe J.M., Moraa E.O., Musyoki J., Murunga N., Mwangi J.N. (2022). Optimization of the SARS-CoV-2 ARTIC Network V4 Primers and Whole Genome Sequencing Protocol. Front. Med..

[44] W-L ProblematicSites_SARS-CoV2—Human-Friendly Version of the Vcf File. https://github.com/W-L/ProblematicSites_SARS-CoV2.

[45] ARTIC Network SARS-CoV-2 Version 4 Scheme Release—Laboratory. https://community.artic.network/t/sars-cov-2-version-4-scheme-release/312.

[46] ARTIC Network SARS-CoV-2 V4.1 Update for Omicron Variant—Laboratory. https://community.artic.network/t/sars-cov-2-v4-1-update-for-omicron-variant/342.

