# Supplementary material for: Evaluation of EPISEQ SARS-CoV-2 and a Fully Integrated Application to Identify SARS-CoV-2 Variants from Several Next-Generation Sequencing Approaches
Source: Viruses. 2022 Jul 29;14(8):1674. doi: 10.3390/v14081674 (PMC9416160; doi:10.3390/v14081674)
Supplement: Supplementary file 1 [file viruses-14-01674-s001.zip › Figure S1.pdf]

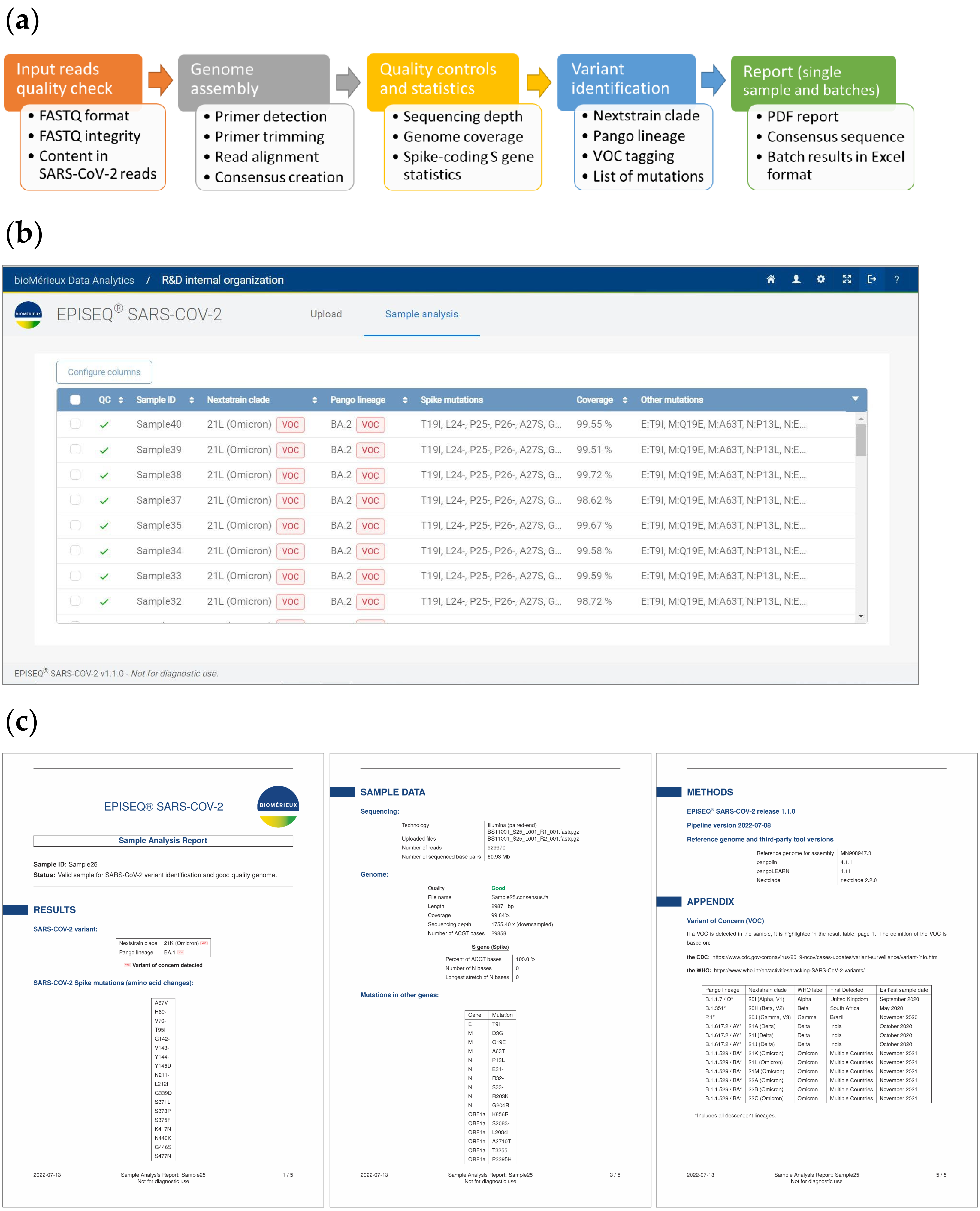

Figure S1: Description of EPISEQ SARS-COV-2 pipeline workflow (**a**), result display (**b**) and sample report (**c**)
