# Supplementary material for: Evaluation of EPISEQ SARS-CoV-2 and a Fully Integrated Application to Identify SARS-CoV-2 Variants from Several Next-Generation Sequencing Approaches
Source: Viruses. 2022 Jul 29;14(8):1674. doi: 10.3390/v14081674 (PMC9416160; doi:10.3390/v14081674)
Supplement: Supplementary file 1 [file viruses-14-01674-s001.zip › Table S1-S3.pdf]

Table S1. Description of the 12 (out of 1362) samples discordant in their Nextstrain clade or Pango lineage assignment between EPISEQ SARS-COV-2 and the reference pipeline.

Dataset	Clade EPISEQ SARS-CoV-2	Clade Reference	Lineage EPISEQ SARS-CoV-2	Lineage Reference
ARTIC v3	20E (EU1)	20E (EU1)	B.1.177	B.1
ARTIC v4	20I (Alpha, V1)	20I (Alpha, V1)	B.1.1.7	Q.6 (alias of B.1.1.7.6)
ARTIC v4	21J (Delta)	21J (Delta)	AY.127 (alias of B.1.617.2.127)	AY.33 (alias of B.1.617.2.33)
ARTIC v4.1	21J (Delta)	21J (Delta)	AY.39 (alias of B.1.617.2.39)	AY.122 (alias of B.1.617.2.122)
ARTIC v4.1	21K (Omicron)	21K (Omicron)	BA.1	BA.1.1
ARTIC v4.1	21K (Omicron)	21K (Omicron)	BA.1	BA.1.1
ARTIC v4.1	21K (Omicron)	21K (Omicron)	BA.1	BA.1.1
ARTIC v4.1	21K (Omicron)	21K (Omicron)	BA.1	BA.1.1
ARTIC v4.1	21K (Omicron)	21K (Omicron)	BA.1	BA.1.1
ARTIC v4.1	21K (Omicron)	21K (Omicron)	BA.1	BA.1.1
ARTIC v4.1	21K (Omicron)	None	BA.1	BA.1
ARTIC v4.1	21K (Omicron)	None	BA.1.1	BA.1.1

Legend: Concordant classifications are highlighted in green; discordant classifications are highlighted in red. Lineage aliases were identified using cov-lineages.org (https://cov-lineages.org/lineage_list.html)

Table S2. Evaluation of SARS-CoV-2 sequencing results with the EPISEQ SARS-COV-2 pipeline (pre-omicron variants; n=21 samples)

			Coverage								Nextclade								Pango							
			Illumina				ONT				Illumina				ONT				Illumina				ONT			
Sample	Ct	VoC	artic_v4.1	articV4	articV3	vss	artic_v4.1	articV4	articV3	vss	artic_v4.1	articV4	articV3	vss	artic_v4.1	articV4	articV3	vss	artic_v4.1	articV4	articV3	vss	artic_v4.1	articV4	articV3	vss
sample1	24,99		99,3	99,8	99,9	99,8	98,6	98,6	99,6	98,3	20A	20A	20A	20A	20A	20A	20A	20A	B.1.221	B.1.221	B.1.221	B.1.221	B.1.221	B.1.221	B.1.221	B.1.221
sample2	30,75		99,4	99,8	99,9	99,8	99,0	99,0	97,1	87,4	20A	20A	20A	20A	20A	20A	20A	20A	B.1.387	B.1.387	B.1.387	B.1.387	B.1.387	B.1.387	B.1.387	B.1.387
sample3	23,06		99,9	99,8	99,9	99,5	99,6	99,6	99,6	98,3	20A	20A	20A	20A	20A	20A	20A	20A	B.1.387	B.1.387	B.1.387	B.1.387	B.1.387	B.1.387	B.1.387	B.1.387
sample4	24,97		99,9	99,9	99,6	99,8	99,6	99,6	98,9	97,4	20A	20A	20A	20A	20A	20A	20A	20A	B.1.147	B.1.147	B.1.147	B.1.147	B.1.147	B.1.147	B.1.147	B.1.147
sample5	24,89		99,9	99,9	100,0	99,8	99,6	99,6	99,6	99,3	20C	20C	20C	20C	20C	20C	20C	20C	B.1	B.1	B.1	B.1	B.1	B.1	B.1	B.1
sample6	30,10		99,9	99,7	99,9	99,7	96,4	98,8	98,9	91,9	20E	20E	20E	20E	20E	20E	20E	20E	B.1.177	B.1.177	B.1.177	B.1.177	B.1.177	B.1.177	B.1.177	B.1.177
sample7	26,61		99,5	99,7	99,9	99,8	98,9	98,9	99,6	98,3	20G	20G	20G	20G	20G	20G	20G	20G	B.1.2	B.1.2	B.1.2	B.1.2	B.1.2	B.1.2	B.1.2	B.1.2
sample8	19,16	Alpha	99,8	99,6	99,9	99,8	99,5	99,5	99,5	98,2	20I	20I	20I	20I	20I	20I	20I	20I	B.1.1.7	B.1.1.7	B.1.1.7	B.1.1.7	B.1.1.7	B.1.1.7	B.1.1.7	B.1.1.7
sample9	17,99	Alpha	99,8	99,7	99,9	99,8	99,5	99,5	99,5	98,2	20I	20I	20I	20I	20I	20I	20I	20I	B.1.1.7	B.1.1.7	B.1.1.7	B.1.1.7	B.1.1.7	B.1.1.7	B.1.1.7	B.1.1.7
sample10	18,71	Alpha	99,8	99,7	99,9	99,8	99,5	99,5	99,5	97,5	20I	20I	20I	20I	20I	20I	20I	20I	B.1.1.7	B.1.1.7	B.1.1.7	B.1.1.7	B.1.1.7	B.1.1.7	B.1.1.7	B.1.1.7
sample11	23,12	Alpha	99,8	99,8	99,9	99,7	99,5	99,5	98,6	98,0	20I	20I	20I	20I	20I	20I	20I	20I	B.1.1.7	B.1.1.7	B.1.1.7	B.1.1.7	B.1.1.7	B.1.1.7	B.1.1.7	B.1.1.7
sample12	27,43	Beta	99,8	99,8	99,3	99,7	99,5	98,6	98,2	98,2	20H	20H	20H	20H	20H	20H	20H	20H	B.1.351	B.1.351	B.1.351	B.1.351	B.1.351	B.1.351	B.1.351	B.1.351
sample13	22,82	Beta	99,8	99,6	99,1	99,2	99,5	99,5	97,9	98,7	20H	20H	20H	20H	20H	20H	20H	20H	B.1.351	B.1.351	B.1.351	B.1.351	B.1.351	B.1.351	B.1.351	B.1.351
sample14	25,09	Gamma	99,9	99,8	99,5	99,8	99,6	99,6	96,8	98,3	20J	20J	20J	20J	20J	20J	20J	20J	P.1.16	P.1.16	P.1.16	P.1.16	P.1.16	P.1.16	P.1.16	P.1.16
sample15	20,02		99,6	99,6	99,5	99,5	99,5	99,5	97,8	97,1	21D	21D	21D	21D	21D	21D	21D	21D	B.1.525	B.1.525	B.1.525	B.1.525	B.1.525	B.1.525	B.1.525	B.1.525
sample16	25,50	Delta	99,4	99,8	99,6	99,8	97,5	98,1	96,7	93,6	21I	21I	21I	21I	21I	21I	21I	21I	AY.66	AY.66	AY.66	AY.66	AY.66	AY.66	AY.66	AY.66
sample17	24,12	Delta	99,8	99,8	99,1	99,8	99,5	99,5	98,6	99,5	21I	21I	21I	21I	21I	21I	21I	21I	AY.9.2	AY.9.2	AY.9.2	AY.9.2	AY.9.2	AY.9.2	AY.9.2	AY.9.2
sample18	15,17	Delta	99,9	99,7	98,6	99,8	99,5	98,8	97,6	99,5	21J	21J	21J	21J	21J	21J	21J	21J	AY.43	AY.43	AY.43	AY.43	AY.43	AY.43	AY.43	AY.43
sample19	missing	Delta	99,2	99,8	98,8	99,1	98,7	97,9	96,8	97,3	21J	21J	21J	21J	21J	21J	21J	21J	AY.5.4	AY.5.4	AY.5.4	AY.5.4	AY.5.4	AY.5.4	AY.5.4	AY.5.4
sample20	29,06	Delta	99,8	99,7	99,3	99,7	99,5	98,8	97,6	98,2	21J	21J	21J	21J	21J	21J	21J	21J	AY.43	AY.43	AY.43	AY.43	AY.43	AY.43	AY.43	AY.43
sample21	27,44	Delta	99,8	99,7	99,1	99,8	98,0	98,1	95,7	94,0	21J	21J	21J	21J	21J	21J	21J	21J	AY.98.1	AY.98.1	AY.98.1	AY.98.1	AY.98.1	AY.98.1	AY.98.1	AY.98.1

	Spike mutation								Other mutations							
	Illumina				ONT				Illumina				ONT			
Sample	artic_v4.1	articV4	articV3	vss	artic_v4.1	articV4	articV3	vss	artic_v4.1	articV4	articV3	vss	artic_v4.1	articV4	articV3	vss
sample1	[S477N, D614G]								N:M234I, N:A376T, ORF1a:M3087I, ORF1b:A176S, ORF1b:P314L, ORF1b:V767L, ORF1b:K1141R, ORF1b:E1184D, ORF3a:A54S, ORF3a:Q57H				- ORF3a:A54S	- ORF3a:A54S	- ORF3a:A54S	- ORF3a:A54S
sample2	[D614G]							-D614G	ORF1a:A3220V, ORF1b:P314L, ORF1b:K798R					-ORF1a:A3220V		
sample3	[D614G]								ORF1a:M1193V, ORF1b:P314L, ORF1b:K798R		+ORF1a:V1169L					
sample4	[D614G]								M:C33F, ORF1a:F2521V, ORF1a:V3718F, ORF1a:A3956V, ORF1b:P314L, ORF1b:V579L, ORF3a:T14I							
sample5	[N188S, D614G]								ORF1a:T265I, ORF1a:T1822I, ORF1b:P314L, ORF3a:A54V, ORF3a:Q57H							
sample6	[A222V, D614G]								N:A220V, ORF1a:D4218N, ORF1b:P314L							
sample7	[D614G]								N:P67S, N:P199L, ORF1a:T265I, ORF1a:L3352F, ORF1b:P314L, ORF1b:N1653D, ORF1b:R2613C, ORF3a:Q57H, ORF3a:G172V, ORF8:S24L							
sample8	[H69-, V70-, Y144-, E484K, N501Y, A570D, D614G, P681H, T716I, S982A, D1118H]								N:D3L, N:R203K, N:G204R, N:S235F, ORF1a:N460Y, ORF1a:T1001I, ORF1a:A1708D, ORF1a:I2230T, ORF1a:S3675-,ORF1a: G3676-, ORF1a:F3677-, ORF1b:D251N, ORF1b:P314L, ORF8:Q27*, ORF8:R52I, ORF8:K68*, ORF8:Y73C, ORF8:L118V, ORF8:F120L							
sample9	[H69-, V70-, Y144-, N501Y, A570D, D614G, P681H, T716I, S982A, D1118H]								N:D3L, N:R203K, N:G204P, N:S235F,ORF1a:T1001I, ORF1a:A1708D, ORF1a:I2230T, ORF1a:S3675-, ORF1a:G3676-, ORF1a:F3677-, ORF1b:P314L, ORF8:Q27*, ORF8:R52I, ORF8:K68*, ORF8:Y73C							
sample10	[H69-, V70-, Y144-, N501Y, A570D, D614G, P681H, T716I, S982A, D1118H]								N:D3L, N:R203K, N:G204R, N:S235F, ORF1a:T1001I, ORF1a:A1708D, ORF1a: I2230T, ORF1a:S3675-, ORF1a:G3676-, ORF1a:F3677-, ORF1b:P314L, ORF7a:P34L, ORF8:Q27*, ORF8:R52I, ORF8:Y73C							
sample11	[H69-, V70-, Y144-, E484K, N501Y, A570D, D614G, P681H, T716I, S982A, D1118H]								N:D3L, N:R203K, N:G204R, N:S235F, ORF1a:K58R, ORF1a:T1001I, ORF1a:A1708D, ORF1a:I2230T, ORF1a:S3675-, ORF1a:G3676-, ORF1a:F3677-, ORF1b:P314L, ORF8:Q27*, ORF8:R52I, ORF8:K68*, ORF8:Y73C							
sample12	[L18F, D80A, D215G, L241-, L242-, A243-, K417N, E484K, N501Y, D614G, A701V]					-A701V			E:P71L, N:T205I, ORF1a:T265I, ORF1a:V649F, ORF1a:K1655N, ORF1a:K3353R, ORF1a:S3675-, ORF1a:G3676-, ORF1a:F3677-, ORF1b:P314L, ORF1b:Q813H, ORF3a:Q57H, ORF3a:S171L							
sample13	[D80A, D215G, L241-, L242-, A243-, K417N, E484K, N501Y, D614G, A701V]						-E484K, -N501Y		E:P71L, N:Q28R, N:T205I, ORF1a:T265I, ORF1a:K1655N, ORF1a:K3353R, ORF1a:S3675-, ORF1a:G3676-, ORF1a:F3677-, ORF1b:P314L, ORF3a:Q57H, ORF3a:S171L, ORF9b:R25G							
sample14	[L18F, T20N, P26S, D138Y, R190S, K417T, E484K, N501Y, D614G, H655Y, T1027I, V1176F]						-E484K, -N501Y		N:P80R, N:R191C, N:R203K, N:G204R, ORF1a:S1188L, ORF1a:K1795Q, ORF1a:S3675-, ORF1a:G3676-, ORF1a:F3677-, ORF1b:P314L, ORF1b:E1264D, ORF3a:I118M, ORF3a:T223I, ORF3a:S253P, ORF8:E92K, ORF9b:Q77E							
sample15	[Q52R, A67V, H69-, V70-, Y144-, K150N, D215Y, E484K, D614G, Q677H, F888L]					-Q52R	-Q52R		E:L21F, M:I82T, N:M1-, N:S2M, N:D3Y, N:A12G, N:T205I, ORF1a:K247N, ORF1a:T2007I, ORF1a:S3675-, ORF1a:G3676-, ORF1a:F3677-, ORF1b:P314F, ORF6:M1-, ORF6:F2M, ORF8:R115L, ORF9b:H9D				-E:L21F	-E:L21F	-E:L21F	-E:L21F
sample16	[T19R, G142D, E156-, F157-, R158G, A222V, L452R, T478K, D614G, P681R, D950N]		-G142D				-G142D,-E156-, -F157-, -R158G, -A222V,	-D614G	M:I82T, N:P6L, N:D63G, N:R203M, N:P207S, N:D377Y, ORF1a:L580F, ORF1a:D981G, ORF1a:G1548S, ORF1a:P1640L, ORF1a:T2846I, ORF1a:A3209V, ORF1a:V3718A, ORF1a:T3750I, ORF1b:P314L, ORF1b:K469N, ORF1b:G662S, ORF1b:P1000L, ORF1b:T2490M, ORF3a:S26L, ORF7a:V24F, ORF7a:T120I, ORF8:D119-, ORF8:F120-, ORF9b:P3S, ORF9b:T60A	+ORF7a:V82A	+ORF7a:V82A	+ORF1b:K2557R, +ORF7a:V82A			-ORF7a:T120I	+ORF7a:V82A
sample17	[T19R, G142D, E156-, F157-, R158G, A222V, L452R, T478K, D614G, P681R, D950N]		-G142D				-G142D		M:I82T, N:D63G, N:R203M, N:D377Y, ORF1a:P1640L, ORF1a:A3209V, ORF1a:V3718A, ORF1a:T3750I, ORF1b:R188Q, ORF1b:P314L, ORF1b:G662S, ORF1b:P1000L, ORF1b:T1540I, ORF3a:S26L, ORF3a:W193R, ORF7a:V82A, ORF7a:T120I, ORF8:D119-, ORF8:F120-, ORF9b:T60A							
sample18	[T19R, G142D, E156-, F157-, R158G, L452R, T478K, D614G, P681R, D950N]		-G142D				-G142D		M:I82T, N:Q9L, N:D63G, N:R203M, N:G215C, N:D377Y, ORF1a:A1306S, ORF1a:K1655N, ORF1a:P2046L, ORF1a:P2287S, ORF1a:V2930L, ORF1a:T3255I, ORF1a:T3646A, ORF1b:P314L, ORF1b:G662S, ORF1b:L829I, ORF1b:P1000L, ORF1b:A1918V, ORF3a:S26L, ORF7a:V82A, ORF7a:T120I, ORF7b:T40I, ORF8:D119-, ORF8:F120-, ORF9b:S6C, ORF9b:T60A							
sample19	[T19R, G142D, E156-, F157-, R158G, L452R, T478K, D614G, P681R, D950N]		-G142D				-G142D		M:I82T, N:D63G, N:R203I, N:G215C, N:S327L, N:D377Y, ORF1a:P309L, ORF1a:A1306S, ORF1a:P1977L,ORF1a:P2287S, ORF1a:V2930L, ORF1a:T3255I, ORF1a:T3646A, ORF1a:F4269L, ORF1b:P314L, ORF1b:G662S,ORF1b: P1000L, ORF1b:A1918V, ORF3a:S26L, ORF7a:V82A,ORF7a:T120I, ORF7b:T40I, ORF8:D119-, ORF8:F120-, ORF9b:T60A	+ORF1a:P2046L	+ORF1a:P2046L	+ORF1a:P2046L				+ORF1a:P2046L
sample20	[T19R, G142D, E156-, F157-, R158G, L452R, T478K, D614G, P681R, D950N]		+A67V, +H69-, +V70-, -G142D				-G142D		M:I82T, N:Q9L, N:D63G, N:R203M, N:G215C, N:D377Y, ORF1a:A1306S, ORF1a:P2046L, ORF1a:P2287S, ORF1a:V2930L, ORF1a:T3255I, ORF1a:T3646A, ORF1b:P314L, ORF1b:G662S, ORF1b:L829I, ORF1b:P1000L, ORF1b:A1918V, ORF3a:S26L, ORF7a:V82A, ORF7a:T120I, ORF7b:T40I, ORF8:D119-, ORF8:F120-, ORF9b:S6C, ORF9b:T60A							
sample21	[T19R, G142D, E156-, F157-, R158G, P251L, L452R, T478K, D614G, P681R, D950N, E1150D]		-G142D				-G142D		M:I82T, N:D63G, N:R203M, N:G215C, N:D377Y, ORF1a:E87D, ORF1a:P1158T, ORF1a:A1306S, ORF1a:L1683I, ORF1a:E1724D, ORF1a:P2046L, ORF1a:P2287S, ORF1a:V2930L, ORF1a:T3255I, ORF1a:T3646A, ORF1b:P314L, ORF1b:G662S, ORF1b:P1000L, ORF1b:A1918V, ORF3a:S26L, ORF3a:L41F, ORF7a:V82A, ORF7a:T120I, ORF7b:T40I, ORF8:A65S, ORF8:D119-, ORF8:F120-, ORF9b:T60A				-ORF7b:T40I	-ORF7b:T40I		- ORF1a:P2046L

Amino acid mutations in spike and other encoded genes are shown relative to the data generated using the Illumina platform with the ARTIC v4.1 kit (empty field: same mutations as in the Illumina/ARTIC v4.1 sequence; fields with "+" or "-" signify the presence or absence of the indicated mutations, respectively. NGS results with genome coverage < 95% are highlighted in grey.

Table S3. Evaluation of SARS-CoV-2 sequencing results with the EPISEQ SARS-COV-2 pipeline (omicron variants; n=19 samples)

sample	Ct	Coverage				Nextclade				Pango				Spike mutation				Other mutations			
		Illumina		ONT		Illumina		ONT		Illumina		ONT		Illumina		ONT		Illumina		ONT	
		artic_v4.1	vss2	artic_v4.1	vss2	artic_v4.1	vss2	artic_v4.1	vss2	artic_v4.1	vss2	artic_v4.1	vss2	artic_v4.1	vss2	artic_v4.1	vss2	artic_v4.1	vss2	artic_v4.1	vss2
sample22	16,86	99,8	92,1	99,5	69,1	21K	21K	21K	21K	BA.1	BA.1	BA.1	no data	[A67V, H69-, V70-, T95I, G142-, V143-, Y144-, Y145D,N211-, L212I, G339D, S371L, S373P, S375F, K417N, N440K, G446S, S477N, T478K, E484A, Q493R, G496S, Q498R, N501Y, Y505H, T547K, D614G, H655Y, N679K, P681H, N764K, D796Y, N856K, Q954H, N969K, L981F]	-N211-, -L212I, -N440K		-N211-, -L212I, -N440K, -G446S, N764K, -D796Y	E:T9I, M:D3G, M:Q19E, M:A63T, N:P13L, N:E31-, N:R32-, N:S33-, N:R203K, N:G204R, ORF1a:K856R, ORF1a:S2083-, ORF1a:L2084I, ORF1a:A2710T, ORF1a:T3255I, ORF1a:P3395H, ORF1a:L3674-, ORF1a:S3675-, ORF1a:G3676-, ORF1a:I3758V, ORF1b:P314L, ORF1b:I1566V, ORF9b:P10S, ORF9b:E27-, ORF9b:N28-, ORF9b: A29-	- ORF1a:L3674-, -ORF1a:S3675-, -ORF1a:G3676-, -ORF1b:I1566V		-E:T9I, -ORF1a:K856R, -ORF1a:A2710T, -ORF1a:L3674-, -ORF1a:S3675-, -ORF1a:G3676-, -ORF1b:I1566V
sample23	15,25	99,9	99,8	99,5	99,4	21K	21K	21K	21K	BA.1	BA.1	BA.1	BA.1	[A67V, H69-, V70-, T95I, G142-, V143-, Y144-, Y145D, N211-, L212I, G339D, S371L, S373P, S375F, K417N, N440K, G446S, S477N, T478K, E484A, Q493R, G496S, Q498R, N501Y, Y505H, T547K, D614G, H655Y, N679K, P681H, N764K, D796Y, N856K, Q954H, N969K, L981F]				E:T9I, M:D3G, M:Q19E, M:A63T, N:P13L, N:E31-, N:R32-, N:S33-, N:R203K, N:G204R, ORF1a:K856R, ORF1a:T1822I, ORF1a:S2083-, ORF1a:L2084I, ORF1a:A2710T, ORF1a:T3255I, ORF1a:P3395H, ORF1a:L3674-, ORF1a:S3675-, ORF1a:G3676-, ORF1a:I3758V,ORF1b: P314L, ORF1b:I1566V, ORF3a:D155Y, ORF9b:P10S, ORF9b:E27-, ORF9b:N28-, ORF9b:A29-			
sample24	17,31	99,8	99,6	99,5	99,4	21K	21K	21K	21K	BA.1	BA.1	BA.1	BA.1	[A67V, H69-, V70-, T95I, G142-, V143-, Y144-, Y145D, N211-, L212I, G339D, S371L, S373P, S375F, K417N, N440K, G446S, S477N, T478K, E484A, Q493R, G496S, Q498R, N501Y, Y505H, T547K, D614G, H655Y, N679K, P681H, N764K, D796Y, N856K, Q954H, N969K, L981F]				{mutations [T9I, D3G, Q19E, A63T, P13L, E31-, R32-, S33-, R203K, G204R, K856R, P1803S, V1887I, S2083-, L2084I, A2710T, M2796T, T3255I, P3395H, L3674-, S3675-, G3676-, I3758V, P314L, I1566V, P10S, E27-, N28-, A29-], gene [E, M, M, M, N, N, N, N, N, N, ORF1a, ORF1a, ORF1a, ORF1a, ORF1a, ORF1a, ORF1a, ORF1a, ORF1a, ORF1a, ORF1a, ORF1a, ORF1b, ORF1b, ORF9b, ORF9b, ORF9b, ORF9b]}			
sample25	17,48	99,8	99,7	99,5	95,5	21K	21K	21K	21K	BA.1	BA.1	BA.1	BA.1	[A67V, H69-, V70-, T95I, G142-, V143-, Y144-, Y145D, N211-, L212I, G339D, S371L, S373P, S375F, K417N, N440K, G446S, S477N, T478K, E484A, Q493R, G496S, Q498R, N501Y, Y505H, T547K, D614G, H655Y, N679K, P681H, N764K, D796Y, N856K, Q954H, N969K, L981F]				E:T9I, M:D3G, M:Q19E, M:A63T, N:P13L, N:E31-, N:R32-, N:S33-, N:R203K, N:G204R, ORF1a:K856R, ORF1a:S2083-, ORF1a:L2084I, ORF1a:A2710T, ORF1a:T3255I, ORF1a:P3395H, ORF1a:L3674-, ORF1a:S3675-, ORF1a:G3676-, ORF1a:I3758V, ORF1b:P314L, ORF1b:T1453I, ORF1b:I1566V, ORF9b:P10S, ORF9b:E27-, ORF9b:N28-, ORF9b:A29-			
sample26	22,77	99,8	99,7	99,5	99,4	21K	21K	21K	21K	BA.1	BA.1	BA.1	BA.1	[A67V, H69-, V70-, T95I, G142-, V143-, Y144-, Y145D, N211-, L212I, G339D, S371L, S373P, S375F, K417N, N440K, G446S, S477N, T478K, E484A, Q493R, G496S, Q498R, N501Y, Y505H, T547K, D614G, H655Y, N679K, P681H, N764K, D796Y, N856K, Q954H, N969K, L981F]				E:T9I, M:D3G, M:Q19E, M:A63T, N:P13L, N:E31-, N:R32-, N:S33-, N:R203K, N:G204R, ORF1a:K856R, ORF1a:T1822I, ORF1a:S2083-, ORF1a:L2084I, ORF1a:P2221H, ORF1a:A2636V, ORF1a:A2710T, ORF1a:T3255I, ORF1a:P3395H, ORF1a:L3674-, ORF1a:S3675-, ORF1a:G3676-, ORF1a:I3758V, ORF1b:P314L, ORF1b:I1566V, ORF1b:G2220E, ORF3a:D155Y, ORF9b:P10S, ORF9b:E27-, ORF9b:N28-, ORF9b:A29-			
sample27	16,12	99,8	99,7	99,5	95,4	21K	21K	21K	21K	BA.1	BA.1	BA.1	BA.1	[A67V, H69-, V70-, T95I, G142-, V143-, Y144-, Y145D, N211-, L212I, G339D, S371L, S373P, S375F, K417N, N440K, G446S, S477N, T478K, E484A, Q493R, G496S, Q498R, N501Y, Y505H, T547K, D614G, H655Y, N679K, P681H, N764K, D796Y, N856K, Q954H, N969K, L981F]				E:T9I, M:D3G, M:Q19E, M:A63T, N:P13L, N:E31-, N:R32-, N:S33-, N:R203K, N:G204R, ORF1a:K856R, ORF1a:T1822I, ORF1a:S2083-, ORF1a:L2084I, ORF1a:A2710T, ORF1a:T3255I, ORF1a:P3395H, ORF1a:L3674-, ORF1a:S3675-, ORF1a:G3676-, ORF1a:I3758V,ORF1b: P314L, ORF1b:I1566V, ORF3a:D155Y, ORF9b:P10S, ORF9b:E27-, ORF9b:N28-, ORF9b:A29-			- ORF1a:T1822I, -ORF1a:A2710T
sample28	19,69	99,8	99,7	99,5	99,2	21K	21K	21K	21K	BA.1	BA.1	BA.1	BA.1	[A67V, H69-, V70-, T95I, G142-, V143-, Y144-, Y145D, N211-, L212I, G339D, S371L, S373P, S375F, K417N, N440K, G446S, S477N, T478K, E484A, Q493R, G496S, Q498R, N501Y, Y505H, T547K, D614G, H655Y, N679K, P681H, N764K, D796Y, N856K, Q954H, N969K, L981F]				E:T9I, M:D3G, M:Q19E, M:A63T, N:P13L, N:E31-, N:R32-, N:S33-, N:R203K, N:G204R, ORF1a:K856R, ORF1a:T1822I, ORF1a:S2083-, ORF1a:L2084I, ORF1a:A2710T, ORF1a:T3255I, ORF1a:P3395H, ORF1a:L3674-, ORF1a:S3675-, ORF1a:G3676-, ORF1a:I3758V,ORF1b: P314L, ORF1b:I1566V, ORF3a:D155Y, ORF9b:P10S, ORF9b:E27-, ORF9b:N28-, ORF9b:A29-			
sample29	16,11	99,8	98,7	99,5	91,9	21K	21K	21K	21K	BA.1.1	BA.1.1	BA.1.1	BA.1.1	[A67V, H69-, V70-, T95I, G142-, V143-, Y144-, Y145D, N211-, L212I, G339D, R346K, S371L S373P, S375F, K417N, N440K, G446S, S477N, T478K, E484A, Q493R, G496S, Q498R, N501Y, Y505H, T547K, D614G, H655Y, N679K, P681H, N764K, D796Y, N856K, Q954H, N969K, L981F]				E:T9I, M:D3G, M:Q19E, M:A63T, N:P13L, N:E31-, N:R32-, N:S33-, N:R203K, N:G204R, ORF1a:R398C, ORF1a:K856R, ORF1a:L890I, ORF1a:S2083-, ORF1a:L2084I, ORF1a:A2710T, ORF1a:T3255I, ORF1a:P3395H, ORF1a:L3674-, ORF1a:S3675-, ORF1a:G3676-, ORF1a:I3758V, ORF1b:P314L, ORF1b:Q866R, ORF1b:I1566V, ORF9b:P10S, ORF9b:E27-, ORF9b:N28-, ORF9b:A29-		-ORF1b:Q866R	-ORF1a:A2710T, -ORF1b:Q866R
sample30	23,00	99,3	99,6	97,9	99,4	21K	21K	21K	21K	BA.1	BA.1	BA.1	BA.1	[A67V, H69-, V70-, T95I, G142-, V143-, Y144-, Y145D, N211-, L212I, G339D, R346S, S371L S373P, S375F, K417N, N440K, G446S, L452R, S477N, T478K, E484A, Q493R, G496S, Q498R, N501Y, Y505H, T547K, D614G, H655Y, N679K, P681H, N764K, D796Y, N856K, Q954H, N969K, L981F]				E:T9I, M:D3G, M:Q19E, M:A63T, M:P13L, N:E31-, N:R32-, N:S33-, N:R203K, N:G204R, N:K856R, ORF1a:P1803S, ORF1a:V1887I, ORF1a:S2083-, ORF1a:L2084I, ORF1a:A2710T, ORF1a:T3255I, ORF1a:P3395H, ORF1a:L3674-, ORF1a:S3675-, ORF1a:G3676-, ORF1a:I3758V, ORF1b:P314L, ORF1b:V464F, ORF1b:I1566V, ORF9b:P10S, ORF9b:E27-, ORF9b:N28-, ORF9b:A29-			
sample31	21,86	98,5	99,6	97,5	98,3	21L	21L	21L	21L	BA.2	BA.2	BA.2	BA.2	[T19I, L24-, P25-, P26-, A27S, G142D, V213G, D405N, R408S, K417N, N440K, S477N, T478K, E484A, Q493R, Q498R, N501Y, Y505H, D614G, H655Y, N679K, P681H, N764K, D796Y, Q954H, N969K]	+G339D, +S371F, +S373P, +S375F, +T376A	-D405N	+G339D, +S371F, +S373P, +S375F, +T376A, -N440K, -S477N, -T478K, -E484A, -Q493R, -Q498R, -N501Y, -Y505H,	E:T9I, M:Q19E, M:A63T, N:P13L, N:E31-, N:R32-, N:S33-, N:R203K, N:G204R, N:S413R, ORF1a:V84-, ORF1a:M85- ORF1a:S135R, ORF1a:T842I, ORF1a:G1307S, ORF1a:L3027F, ORF1a:T3090I, ORF1a:L3201F, ORF1a:T3255I, ORF1a:P3395H, ORF1a:F3604L, ORF1a:S3675-, ORF1a:G3676-, ORF1a:F3677-, ORF1b:P314L, ORF1b:R1315C, ORF1b:I1566V, ORF1b:T2163I, ORF3a:T223I, ORF6:D61L, ORF9b:P10S, ORF9b:E27-, ORF9b:N28-, ORF9b:A29-			
sample32	18,27	98,7	99,6	98,4	96,5	21L	21L	21L	21L	BA.2	BA.2	BA.2	BA.2	[T19I, L24-, P25-, P26-, A27S, G142D, V213G, D405N, R408S, K417N, N440K, S477N, T478K, E484A, Q493R, Q498R, N501Y, Y505H, D614G, H655Y, N679K, P681H, N764K, D796Y, Q954H, N969K]	+G339D, +S371F, +S373P, +S375F, +T376A	-D405N	+G339D, +S371F, +S373P, +S375F, +T376A, -N440K, -S477N, -T478K, -E484A, -Q493R, -Q498R, -N501Y, -Y505H,	E:T9I, M:Q19E, M:A63T, N:P13L, N:E31-, N:R32-, N:S33-, N:R203K, N:G204R, N:S413R, ORF1a:S135R, ORF1a:T842I, ORF1a:G1307S, ORF1a:L3027F, ORF1a:T3090I, ORF1a:L3201F, ORF1a:T3255I, ORF1a:P3395H, ORF1a:S3675-, ORF1a:G3676-, ORF1a:F3677-, ORF1b:T76I, ORF1b:P314L, ORF1b:R1315C, ORF1b:I1566V, ORF1b:T2163I, ORF3a:T223I, ORF6: D61L, ORF9b:P10S, ORF9b:E27-, ORF9b:N28-, ORF9b:A29-			
sample33	20,64	99,6	99,5	98,4	94,5	21L	21L	21L	21L	BA.2	BA.2	BA.2	BA.2	[T19I, L24-, P25-, P26-, A27S, G142D, V213G, G339D, S371F, S373P, S375F, T376A, D405N, R408S, K417N, N440K, S477N, T478K, E484A, Q493R, Q498R, N501Y, Y505H, D614G, H655Y, N679K, P681H, N764K, D796Y, Q954H, N969K]	-Q498R, -N501Y, -Y505H	-G339D, -S371F, -S373P, -S375F, -T376A, -D405N	-N440K, -S477N, -T478K, -E484A, -Q493R, -Q498R, -N501Y, -Y505H,	E:T9I, M:Q19E, M:A63T, N:P13L, N:E31-, N:R32-, N:S33-, N:R203K, N:G204R, N:S413R, ORF1a:S135R, ORF1a:T842I, ORF1a:G1307S, ORF1a:L3027F, ORF1a:T3090I, ORF1a:L3201F, ORF1a:T3255I, ORF1a:P3395H, ORF1a:S3675-, ORF1a:G3676-, ORF1a:F3677-, ORF1b:P314L, ORF1b:R1315C, ORF1b:I1566V, ORF1b:T2163I, ORF3a:T223I, ORF6:D61L, ORF9b:P10S, ORF9b:E27-, ORF9b:N28-, ORF9b:A29-			
sample34	28,92	99,6	97,3	98,8	97,0	21L	21L	21L	21L	BA.2	BA.2	BA.2	BA.2	[T19I, L24-, P25-, P26-, A27S, G142D, V213G, G339D, S371F, S373P, S375F, T376A, D405N, R408S, K417N, N440K, S477N, T478K, E484A, Q493R, Q498R, N501Y, Y505H, D614G, H655Y, N679K, P681H, N764K, D796Y, Q954H, N969K]	-N440K, -S477N, -T478K, -E484A, -Q493R, -Q498R, -N501Y, -Y505H		'-N440K, -S477N, -T478K, -E484A, -Q493R, -Q498R, -N501Y, -Y505H	E:T9I, M:Q19E, M:A63T, N:P13L, N:E31-, N:R32-, N:S33-, N:R203K, N:G204R, N:S413R, ORF1a:S135R, ORF1a:T842I, ORF1a:G1307S, ORF1a:L3027F, ORF1a:T3090I, ORF1a:L3201F, ORF1a:T3255I, ORF1a:P3395H, ORF1a:S3675-, ORF1a:G3676-, ORF1a:F3677-, ORF1b:P314L, ORF1b:R1315C, ORF1b:I1566V, ORF1b:T2163I, ORF3a:T223I, ORF6:D61L, ORF9b:P10S, ORF9b:E27-, ORF9b:N28-, ORF9b:A29-			
sample35	15,59	99,7	95,8	96,8	97,0	21L	21L	21L	21L	BA.2	BA.2	BA.2	BA.2	[T19I, L24-, P25-, P26-, A27S, G142D, V213G, G339D, S371F, S373P, S375F, T376A, D405N, R408S, K417N, N440K, S477N, T478K, E484A, Q493R, Q498R, N501Y, Y505H, D614G, H655Y, N679K, P681H, N764K, D796Y, Q954H, N969K]	-N440K, -S477N, -T478K, -E484A, -Q493R, -Q498R, -N501Y, -Y505H	-G339D, -S371F, -S373P, -S375F, -T376A, -D405N	-N440K, -S477N, -T478K, -E484A, -Q493R, -Q498R, -N501Y, -Y505H	E:T9I, M:Q19E, M:A63T, N:P13L, N:E31-, N:R32-, N:S33-, N:R203K, N:G204R, N:S413R, ORF1a:S135R, ORF1a:T842I, ORF1a:G1307S, ORF1a:L3027F, ORF1a:T3090I, ORF1a:L3201F, ORF1a:T3255I, ORF1a:P3395H, ORF1a:S3675-, ORF1a:G3676-, ORF1a:F3677-, ORF1b:P314L, ORF1b:R1315C, ORF1b:I1566V, ORF1b:T2163I, ORF3a:T223I, ORF6:D61L, ORF9b:P10S, ORF9b:E27-, ORF9b:N28-, ORF9b:A29-			
sample36	21,78	99,4	98,5	97,5	96,1	21L	21L	21L	21L	BA.2	BA.2	BA.2	BA.2	[T19I, L24-, P25-, P26-, A27S, G142D, V213G, G339D, D405N, R408S, K417N, N440K, S477N, T478K, E484A, Q493R, Q498R, N501Y, Y505H, D614G, H655Y, N679K, P681H, N764K, D796Y, Q954H, N969K]	+S371F, +S373P, +S375F, +T376A, -N440K, -S477N, -T478K, -E484A, -Q493R, -Q498R, -N501Y, -Y505H,	-G339D, -D405N	+S371F, +S373P, +S375F, +T376A, -N440K, -S477N, -T478K, -E484A, -Q493R, -Q498R, -N501Y, -Y505H	E:T9I, M:Q19E, M:A63T, N:P13L, N:E31-, N:R32-, N:S33-, N:R203K, N:G204R, N:S413R, ORF1a:S135R, ORF1a:T842I, ORF1a:G1307S, ORF1a:A2529V, ORF1a:L3027F, ORF1a:T3090I, ORF1a:L3201F, ORF1a:T3255I, ORF1a:P3395H, ORF1a:S3675-, ORF1a:G3676-, ORF1a:F3677-, ORF1b:P314L, ORF1b:R1315C, ORF1b:I1566V, ORF1b:T2163I, ORF3a:H78Y, ORF3a:T223I, ORF6:D61L, ORF9b:P10S, ORF9b:E27-, ORF9b:N28-, ORF9b:A29-			
sample37	21,32	98,6	97,6	94,3	97,4	21L	21L	21L	21L	BA.2	BA.2	BA.2	BA.2	[T19I, L24-, P25-, P26-, A27S, G142D, V213G, D405N, R408S, K417N, N440K, S477N, T478K, E484A, Q493R, Q498R, N501Y, Y505H, D614G, H655Y, N679K, P681H, N764K, D796Y, Q954H, N969K]	+G339D, +S371F, +S373P, +S375F, +T376A, -N440K, -S477N, -T478K, -E484A, -Q493R, -Q498R, -N501Y, -Y505H	-D405N	+G339D, +S371F, +S373P, +S375F, +T376A, -N440K, -S477N, -T478K, -E484A, -Q493R, -Q498R, -N501Y, -Y505H,	E:T9I, M:Q19E, M:A63T, N:P13L, N:E31-, N:R32-, N:S33-, N:R203K, N:G204R, N:S413R, ORF1a:S135R, ORF1a:T842I, ORF1a:G1307S, ORF1a:L3027F, ORF1a:T3090I, ORF1a:L3201F, ORF1a:T3255I, ORF1a:P3395H, ORF1a:S3675-, ORF1a:G3676-, ORF1a:F3677-, ORF1b:P314L, ORF1b:R1315C, ORF1b:I1566V, ORF1b:T2163I, ORF3a:T223I, ORF6:D61L, ORF9b:P10S, ORF9b:E27-, ORF9b:N28-, ORF9b:A29-		- ORF3a:T223I	
sample38	15,02	99,7	99,7	99,4	98,3	21L	21L	21L	21L	BA.2	BA.2	BA.2	BA.2	[T19I, L24-, P25-, P26-, A27S, G142D, V213G, G339D, S371F, S373P, S375F, T376A, D405N, R408S, K417N, N440K, S477N, T478K, E484A, Q493R, Q498R, N501Y, Y505H, D614G, H655Y, N679K, P681H, N764K, D796Y, Q954H, N969K]	- N440K		-N440K, -S477N, -T478K, -E484A, -Q493R, -Q498R, -N501Y, -Y505H	E:T9I, M:Q19E, M:A63T, N:P13L, N:E31-, N:R32-, N:S33-, N:R203K, N:G204R, N:S413R, ORF1a:S135R, ORF1a:T842I, ORF1a:G1307S, ORF1a:L3027F, ORF1a:T3090I, ORF1a:L3201F, ORF1a:T3255I, ORF1a:P3395H, ORF1a:S3675-, ORF1a:G3676-, ORF1a:F3677-, ORF1b:P314L, ORF1b:R1315C, ORF1b:I1566V, ORF1b:T2163I, ORF3a:T223I, ORF6:D61L, ORF9b:P10S, ORF9b:E27-, ORF9b:N28-, ORF9b:A29-			
sample39	20,91	99,5	98,1	98,4	96,0	21L	21L	21L	21L	BA.2	BA.2	BA.2	BA.2	[T19I, L24-, P25-, P26-, A27S, G142D, V213G, G339D, S373P, S375F, T376A, D405N, R408S, K417N, N440K, S477N, T478K, E484A, Q493R, Q498R, N501Y, Y505H, D614G, H655Y, N679K, P681H, A688V, N764K, D796Y, Q954H, N969K]	+S371F, -S477N, -T478K, -E484A, -Q493R, -Q498R, -N501Y, -Y505H	-G339D, -S373P, -S375F, -T376A, -D405N	+S371F, -N440K, -S477N, -T478K, -E484A, -Q493R, -Q498R, -N501Y, -Y505H	E:T9I, M:Q19E, M:A63T, N:P13L, N:E31-, N:R32-, N:S33-, N:R203K, N:G204R, N:S413R, ORF1a:V84-, ORF1a:M85- ORF1a:S135R, ORF1a:T842I, ORF1a:V874I, ORF1a:D1088G, ORF1a:G1307S, ORF1a:L3027F, ORF1a:T3090I, ORF1a:L3201F, ORF1a:T3255I, ORF1a:P3395H, ORF1a:S3675-, ORF1a:G3676-, ORF1a:F3677-, ORF1b:P314L, ORF1b:R1315C, ORF1b:I1566V, ORF1b:T2163I, ORF3a:T223I, ORF6:D61L, ORF9b:P10S, ORF9b:E27-, ORF9b:N28- , ORF9b:A29-	-ORF1a:D1088G	-ORF1a:V84-, -ORF1a:M85-	-ORF1a:D1088G
sample40	19,42	99,6	99,5	97,5	98,3	21L	21L	21L	21L	BA.2	BA.2	BA.2	BA.2	[T19I, L24-, P25-, P26-, A27S, G142D, V213G, G339D, S371F, S373P, S375F, T376A, D405N, R408S, K417N, N440K, S477N, T478K, E484A, Q493R, Q498R, N501Y, Y505H, D614G, H655Y, N679K, P681H, N764K, D796Y, Q954H, N969K]		-G339D, -S371F, -S373P, -S375F, -T376A, -D405N	-N440K, -S477N, -T478K, -E484A, -Q493R, -Q498R, -N501Y, -Y505H	E:T9I, M:Q19E, M:A63T, N:P13L, N:E31-, N:R32-, N:S33-, N:R203K, N:G204R, N:S413R, ORF1a:S135R, ORF1a:T842I, ORF1a:G1307S, ORF1a:L3027F, ORF1a:T3090I, ORF1a:L3201F, ORF1a:T3255I, ORF1a:P3395H, ORF1a:S3675-, ORF1a:G3676-, ORF1a:F3677-, ORF1b:P314L, ORF1b:R1315C, ORF1b:I1566V, ORF1b:T2163I, ORF3a:T223I, ORF6:D61L, ORF9b:P10S, ORF9b:E27-, ORF9b:N28-, ORF9b:A29-			

Amino acid mutations in spike and other encoded genes are shown relative to the data generated using the Illumina platform with the ARTIC v4.1 kit (empty field: same mutations as in the Illumina/ARTIC v4.1 sequence; fields with "+" or "-" signify the presence or absence of the indicated mutations, respectively. NGS results with genome coverage < 95% are highlighted in grey. One NGS data with low genome coverage (69.1%) could not be assigned a Pango lineage (yellow field).
